# Three-dimensional runout characterisation for rotationally symmetric components

**DOI:** 10.1038/s44172-025-00354-0

**Published:** 2025-02-12

**Authors:** Christopher G. Tompkins, Luke D. Todhunter, Harald Gottmann, Christoph Rettig, Robert Schmitt, Jochen Wacker, Samanta Piano

**Affiliations:** 1 Manufacturing Metrology Team, Faculty of Engineering, University of Nottingham, Nottingham, NG8 1BB UK; 2 Dentsply Sirona, 64625 Bensheim, Germany

**Keywords:** Mechanical engineering, Scientific data, Characterization and analytical techniques

## Abstract

Rotationally symmetric components (such as gears and axels) are ubiquitous to modern devices, and their precision manufacture is necessary to keep costs and manufacture time down, as well as reduce waste and possibly hazardous component failure. The manufacturing errors, which affect the shape in the rotation axis, are grouped together into the common term “runout". Here we present a potential updated standard for characterising the runout of rotationally symmetric machined parts in three-dimensions, and evaluated using virtual instrumentation, enabling an accurate characterisation of the three dimensional (3D) surface deformation of a part from minimal surface information. For any 3D characterisation method to be widely adopted by the science, technology, engineering, and mathematics community, it must be fully compatible with previous methods and standards. As such, the proposed method produces a 3D runout vector based on four standard profile measurements. To evaluate the efficacy of the proposed runout method, a technique for evaluating the errors of commonly used virtual instruments has been developed. This evaluation technique produces a single-valued quantification of the deviation of the instrument outputs compared to the input parameters, decoupled from the errors on the instrument itself.

## Introduction

The accurate fabrication of rotating mechanical systems is a key factor in modern manufacture and engineering. Almost all vehicles, mechanical tools, cooling systems, and pumps rely on rotational elements to function^1–4^. Therefore, accurate and repeatable manufacture of these components is important, as defects can negatively affect multiple factors when the part is in use. Defects can cause increased wear, and lower the lifetime of parts, due to varying stress in the regions where components mesh^1^. These components can be difficult and expensive to replace^5^, and their failure can be hazardous when included in high-power systems. These systems may also no longer transmit energy only along the desired direction, vibrating instead. This not only adds noise, which can add to the hazards associated with large machinery^6^ and adds stress to surrounding components, but also wastes a notable portion of input power/fuel over the lifetime of the part. Vibration itself, due to numerous factors, has been demonstrated to increase fuel consumption in aircraft by up to 20%^2^, thereby impacting their efficiency. When rotating parts are used to make other components, such as drilling holes, these errors propagate into the new part, increasing the size of the borehole beyond what is desired^3^, leading to ill-fitting and mismatched components.

The errors on a rotationally symmetric part may be grouped into two classes: runout and roundness errors. Runout is a measure of how close the centre of an object rotates to the axis of rotation^7^, while roundness is a measure of the shape of the part itself^8^. These errors may again be broken down into the physical properties of the part which govern them: part tilt, concentricity, and lobing. Traditionally, these physical features are not contextualised; runout and roundness is measured only as the scalar deviation using the maximum and minimum measured radii of the part, without links to the govening geometry^7,9,10^. This measure is useful for determining if a part is overall in tolerance (and whether the part needs to be rejected/retooled), however the lack of information about the directionality of these dimensional errors may lead to an unintended increase/decrease in errors when the part is used or installed. For example, a chuck and shaft may both have axial runout within acceptable tolerance, but combining them without knowing the orientation of these lobes may result in the errors combining additively (becoming out of tolerance), whereas a slight rotation of one of these parts could instead result in a part with an overall error smaller than the individual errors on both parts. Characterisation of these surfaces in three dimensions (3D) is therefore favourable, resulting in more precise control of the overall product dimensionality. To do so, it is sensible to consider the current measurement standards and how they can be extended, and why they have not been previously.

The current 1D standards of roundness measurement have remained effectively unchanged since their implementation in 1985^11^, before current digital analysis methods became widespread or affordable. Their limitations were transferred over from by-hand methods, and have been maintained for ease of use and traceability. However, technological advancements within the current automation and industry 4.0 framework mean that extending these standard beyond simple magnitude measurements, maintaining backwards compatibility and using the already increasing computation of smart manufacturing, has now become both feasible and desirable in a large number of applications^2,3,12–14^. An addition of more physically representative information in the measurement, kept concise, would provide more knowledge of the measurand and allow for techniques like smart pairing of components using available machinery, instead of resource-heavy retooling/rejection. Recent reviews of the current smart-manufacturing landscape highlight how current methodologies may not be suited to automated processes such as this. They instead require more data driven approaches to allow for proper machine control, instead of the intuitive leaps humans may make, but with potential decreases in financial/time cost in implementing them^15^. Of course, maintaining traceability with these new techniques then becomes an important challenge - the new methods must be compatible with the old. Outlined in this paper is, to the authors knowledge, the first proposed extension to these standards: a fully backwards-compatible extension which characterises runout in 3D, as a single-valued runout vector. This method is tested and characterised to determine the limits of the physical features it can measure, using another modern manufacturing tool: a virtual instrument.

Virtual instruments and simulations are common methods of evaluating techniques within the STEM community, although it can be difficult to both quantify the results in a concise way and extract any errors in the instrument from errors in any analysis performed on it^16–20^. In this work, a novel alternative method to evaluating the errors on these instruments was developed. This is based on analysing the mean absolute error between the input parameters used to define a simulation, and their equivalents extracted from the output of the instrument, for a well characterised test component. This has allowed the efficacy of the instrument to be quantified, separately from any prior simulation steps, or any analysis performed on the data afterwards. This method produces a single-valued result which allowed all properties to be easily quantified. The proposed runout model is defined in the section titled “The proposed 3D runout model”, and the equations which define the topographies of the simulated parts used to test it in the section titled “Modelling virtual parts”. A breakdown of the virtual instrument and the methods used to analyse the model is then given in the section titled “Experimental methods of model validation”, with the final results of the analysis detailed in the “Results” section.

## Methods

### Nomenclature

- *h* = the height of a part
- *ρ* = the minimum element size/resolution of the simulation
- *r* = external radial profile of a part
- *θ* = polar angle
- *δθ* = a rotation along the *θ* direction
- *n* = number of lobes in a parts profile
- ${R}_{\min }$ = minimum radius of circle circumscribing the radial profile
- ${R}_{\max }$ = maximum radius of circle circumscribing the radial profile
- *δr* = asperity height above/below mean radial profile position
- *ϕ*(*x*) = distribution of asperity heights, e.g. Gaussian or Weibull
- Δ*r* = surface roughness above above/below mean radial profile position
- *F*^−1^[*x*] = the inverse Fourier transform of distribution *x*
- MAE = Mean Absolute Error
- Component = A piece that is measured in the system, be it the target of the measurement or part of the device which takes the reading.
- Part = The target component of the measurement, and the component that the user intends to find the runout of
- Mandrel = The part of the system that the part is mounted on, in order to couple it to the system and rotate it. This will also have its own runout, which is decoupled from the runout of the part.

The work presented below is a summary of an academic-industrial collaboration to develop a method of runout analysis for rotationally-symmetric objects. This method contains additional spatial and geometric information of the measurand, directly linking the measurement to its physical properties, to allow for a more physically-representative measurement. The full development pipeline of the work is presented in the Supplementary Material (Item 1).

The first methodology section in this work details the proposed 3D runout method, listing the measurement process and how the 3D measure is produced from four ISO-complaint measurements by defining a set of runout vectors. The section also proceeds to explain the additional relevant measurement quantities that other methods cannot measure: part orientation, part tilt, and runout direction. Following the 3D runout definition, a virtual instrument is presented.

The virtual instrument was developed by identifying the relevant physical quantities in the current runout and roundness standards^8,9^, namely: surface lobing, surface roughness, axial runout, and concentricity. Bulk experimental data are then aggregated and developed into a generic rotationally-symmetric part, where relevant parameters are varied based on previous data to iterate over different parts.

The analysis process for these virtual parts is then detailed, using a process similar to that in previous works, where large experimental datasets are analysed^21–24^. In this proposed analysis process, a component generated from the measured runout parameters is compared to the initial measurand, and conformance between the two is evaluated. If the 3D runout method accurately captures the component characteristics (within some quantified uncertainty in the instrument), then the method will replicate the surface and the two components will be functionally identical.

The conformance in then analysed for a variety of parameters and the limiting properties of the measurement technique identified. These are then summarised, with a recommended measurement protocol detailed afterwards.

### The proposed 3D runout model

The proposed model builds on simple magnitude-only measurement standards, to a more comprehensive 3D method which provides spatial-geometric information. Only the overall runout of the part is considered in this work, measuring from one end of the part to the other, to determine the net runout across the length of a parts fixturing. This runout model extends the standard definition of runout^7^ by giving it magnitude and direction - a runout vector. By extending the ISO-compliant methods in this way, combining multiple already standardised measurements to produce a vector with additional data, all currently recoverable measurable features may be measured with the same accuracy and reliability. Traceability is, crucially for upgrading facilities, therefore fully maintained by this runout vector. This vector is defined in cylindrical polar coordinates, giving a magnitude in the $\hat{r}$ axis and a direction in the $\hat{\theta }$ axis. The $\hat{z}$ axis is defined normal to the plane of rotational symmetry. To define the two measurement points required for a runout vector, two radial profile scans of a part are required, and the centres of each of these scans measured. The centres of these scans are defined in Cartesian coordinates ((*x*_1_, *y*_1_, *z*_1_), (*x*_2_, *y*_2_, *z*_2_)), and it is their position (relative to the centre of the coordinate axes) which is used to define the runout magnitude and angle. This is achieved by a cylindrical polar coordinate conversion: the coordinate axis is defined relative to the centre of rotation, i.e. the centre of the system the component is mounted in and not the component itself, as the system is the source of the rotation and the component may have some concentric runout.

**Fig. 1:**
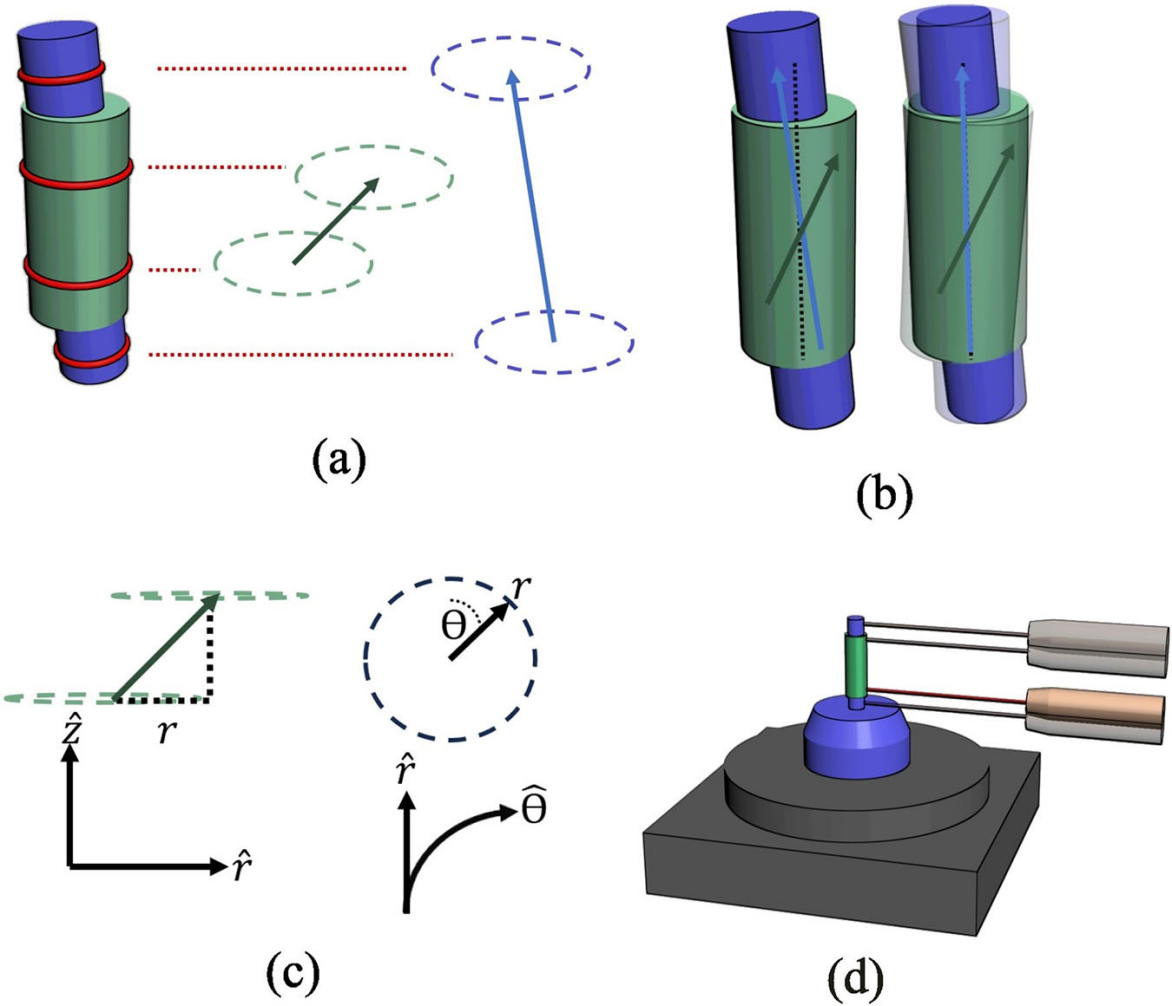
The proposed method of defining three-dimensional runout. In **a** the part in question (green) is mounted on a mandrel (blue), about which it is rotated. The four measurement positions are highlighted in red. The runout vectors of each component are then visualised. In **b** the runout of the mandrel is subtracted from the part, leaving the runout of the part defined only in the coordinate axes. Silhouettes of the assembly have been added to illustrate the transform. **c** shows how the runout vector is defined in the coordinate axes ($\hat{r}$ and $\hat{\theta }$), with magnitude *r* and direction *θ*. $\hat{z}$ is the final direction which defines the cylindrical polar coordinate system, and is normal to the plane of rotation. Finally, in **d** an example of the potential hardware set-up for this is shown. The part and mandrel are now mounted on a rotation stage (grey), with a sensor (orange) directed at one of the four measurement zones, with the remaining 3 zones silhouetted.

In real systems, the mounting fixture will also potentially have measurable runout. Measuring relative to the fixturing negates this, however to do so, the runout of the fixture must be subtracted from that of the part. Overall, this results in a four-parameter measurement protocol, illustrated in Fig. 1.

As this method requires knowledge of the orientation of the parts, standard methods of measuring roundness^25,26^ have been deemed insufficient to evaluate the profiles, as these methods only capture the magnitude of the deviation. Instead, to capture the magnitude and orientation, an updated least-squares regression model based on the polar expression for a lobed circle has been developed:

1 $$r(\theta )=\frac{{R}_{\min }{R}_{\max }}{\sqrt{{\left({R}_{\min }\cos \left(\frac{n\theta +\delta \theta }{2}\right)\right)}^{2}+{\left({R}_{\max }\sin \left(\frac{n\theta +\delta \theta }{2}\right)\right)}^{2}}}.$$

Here *r* is the external radius of the part at a given angle *θ* (with some rotation along the $\hat{\theta }$ direction, *δθ*), *n* is the number of lobes, ${R}_{\min }$ is the minimum radius of the profile, and ${R}_{\max }$ is the maximum profile radius. Equation (1) defines the radial cross section of each rotationally symmetric component, assuming no surface roughness, which must be included separately. The standard measure of roundness is recovered as ${R}_{\max }-{R}_{\min }$, and values *n* and *δθ* capture the orientation.

### Modelling virtual parts

It has long been been established that, within the physical constrains of non-elastic and non-environmentally aged surfaces, there are a small number of parameters which define their shape. These parameters are commonly grouped into surface roughness and surface profile parameters (further broken down into runout and roundness). Since these physical features are those that cause runout and roundness deviations, it is these that have been aggregated to form the instrument, and that it aims to measure with the proposed 3D runout method. To keep applicability broad, no component-type specific (e.g. gear) features are considered, instead the components are kept as general cylinders with runout and roundness perturbations added to their shape. These perturbations cover the complete set of runout, roundness, and surface roughness features expected on physical parts from previous experimental work and known measuremnt protocols^3,10,27–31^.

The virtual components themselves were defined on-grid; a 3D byte array was built, where each complete/empty byte represented the surface/free space (respectively). The limit of this array is  ≈ 24 GB, as the simulation required GPU acceleration and this was the maximum GPU memory available. For parts simulated as 1 mm − 2 mm in diameter and up to 10 mm in height, the number of elements in the array allowed a resolution equivalent to 5*μm*. This resolution is also considered suitable, as it is close to the optical diffraction limit and therefore comparable to real measurement systems. Each physical quantity that represents the object’s shape was then mathematically defined, and added to the grid by drawing the outer profile and filling the part inward. The pipeline which describes all constituent steps is presented in the remainder of this section.

When considering the outer profiles of the 2D slices which define the components to be circles with a set of deformations added, it is possible to group these deformations into two regimes which may be applied to each circle: positional offsets (e.g. tilt), *θ*-dependent deformation (e.g lobing). Starting from a perfect cylindrical cross-section, *θ*-dependent deformations were added first: These were grouped into lobing (caused by machining errors in real components), and surface roughness (common to all surfaces). Lobing was once again defined using Eq. (1). Following this, surface roughness was added to the part.

A common method to include microscopic surface roughness onto surfaces is based on the Greenwood model, where surface roughness is described only by a statistical distribution of asperity heights above a mean position^32,33^. In this case, this mean position is the profile *r* calculated in Eq. (1) above. If the generated distribution of asperity heights (*ϕ*(*δr*)), where *δr* is the asperity height with respect to a mean height (Δ*r*), then it may be assumed that the surface roughness above the mean height is simply the inverse Fourier transform of this spectrum of heights^27,33^:

2 $$\Delta r={F}^{-1}\left[\phi (\delta r)\right].$$

In this work, a Gaussian distribution of asperity heights was used, as this is common to freshly machined surfaces^28,29^.

If the mean position is the radius previously calculated in Eq. (1), then the profile may now be described as:

3 $$r(\theta ,\delta r)=r(\theta )+\Delta r.$$

After each individual cross section is altered in shape, it was then altered in position, to add the remaining runout features. The features that were considered for positional offsets were concentricity and tilt - both due to machining flaws. Concentricity was added by recentring the component on the grid, and tilt was applied by rotating the entire array volume. Both transforms could be performed by simple 3D matrix transform algorithms, already inbuilt into MATLAB.

**Fig. 2:**
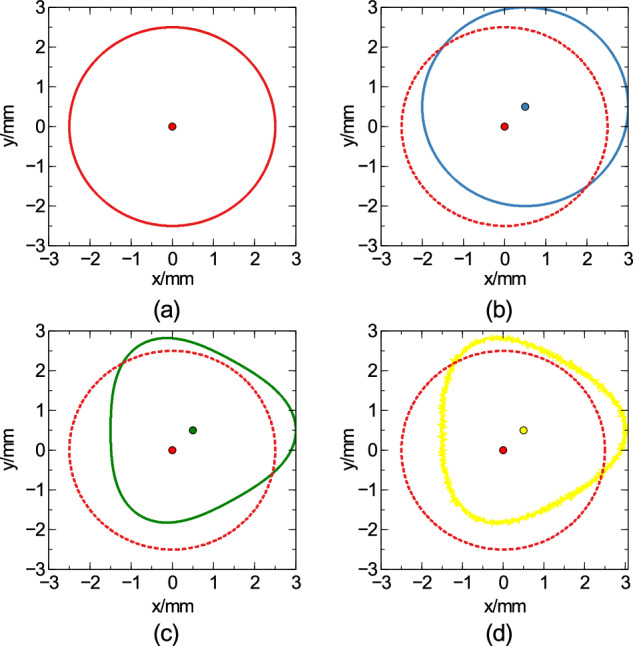
An example two-dimensional cross section of a three-dimensional component, as various surface features are added to the model. The ideal, perfectly cylindrical case (red) is in **a** and shown throughout the subsequent images as a dotted red profile. The centre of the component is indicated by a single point. In **b** an offset has been added to the (blue) component, such that it is no longer centred at the origin of the plane. This could also be due to shear of the component, and cannot be distinguished from just one profile, needing a three-dimensional runout measure. In **c** the (green) component is now lobed (with *n* = 3 lobes), due to some tool oscillation during lathing. Finally, Gaussian surface roughness has been added in **d** (yellow).

With all these features in place, a generic surface with runout features was fully built into a virtual instrument, with tunable parameters to mimic different components. An example of what a profile scan of one of these components would look like, as the component-profile evolves with each added parameter, is shown in Fig. 2.

### Experimental methods of model validation

The presented method of defining a virtual component was applied twice: once for the part, and once for the mandrel it was mounted on, given imperfect mounting is likely in real systems. This virtual part was then scanned by keeping a virtual distance sensor at a fixed position outside of the measuring volume, and rotating the volume about its centre (not necessarily the centre of the part or mandrel). The distance to the part was measured, and the part rotated again. This was completed 360 times, at a 1° rotation per increment. the sensor was then moved to a new height, and the process repeated. The sensor was positioned at four heights during this rotation, two of which would scan the part, and two of which would scan the mandrel. The output of this process was four profile scans which could then be used to generate runout vectors, as described above.

Throughout all virtual experiments, the dimensions of the components were kept the same: a part of minimum radius 2 mm and height 7.5 mm, and a mandrel of minimum radius 1 mm and height 10 mm. These were chosen as they are of the same order of magnitude as common small machined components. In the first set of experiments, only one property of the part is varied at a time (tilt angle, offset, lobing, or surface roughness), to determine which individual parameters have a strong effect on the runout algorithms’ ability to recover all initial parameters. The mandrel is kept as an ideal cylinder in this case, effectively assuming perfect manufacturing. Once these key parameters have been identified, a set of experiments are made where they are varied in pairs, exploring how the combination of multiple key parameters affect the measurement accuracy. In this case, features may be a combination of part and mandrel parameters, such as a tilted part on a tilted mandrel.

As there is a five-dimensional data-space, the conformance of each individual parameter cannot be directly plotted in any concise manner, although these may also be found in the data repository for individualised inquiries^21–24^. For this reason the mean absolute error (MAE) of the residuals between input (*I*) and output (*O*) datasets are calculated following works by^21–24^:

4 $${{{\rm{MAE}}}}=\frac{\mathop{\sum }_{{{{\rm{i}}}}}^{{{{\rm{n}}}}}\left\vert {I}_{{{{\rm{i}}}}}-{O}_{{{{\rm{i}}}}}\right\vert }{n\overline{I}},$$

where i is each element within the set. In this case, the datasets are profiles scans of a generated surface with the parameters: tilt, part offset, lobe amplitude, lobe count, and mean asperity height. Two profiles are therefore not identical unless all parameters which define them are identical. To generate the two sets of profiles, 3D part-mandrel assemblies are generated and analysed with the virtual instrument, one with the input parameters (*I*), and one with the output parameters (*O*). The input parameters are user defined, and the output parameters were obtained by scanning the initial part and then recovering the runout/roundess parameters with the proposed 3D runout model, then using these to generate a new part. This new part is then measured, to generate new profile scans for comparison.

**Fig. 3:**
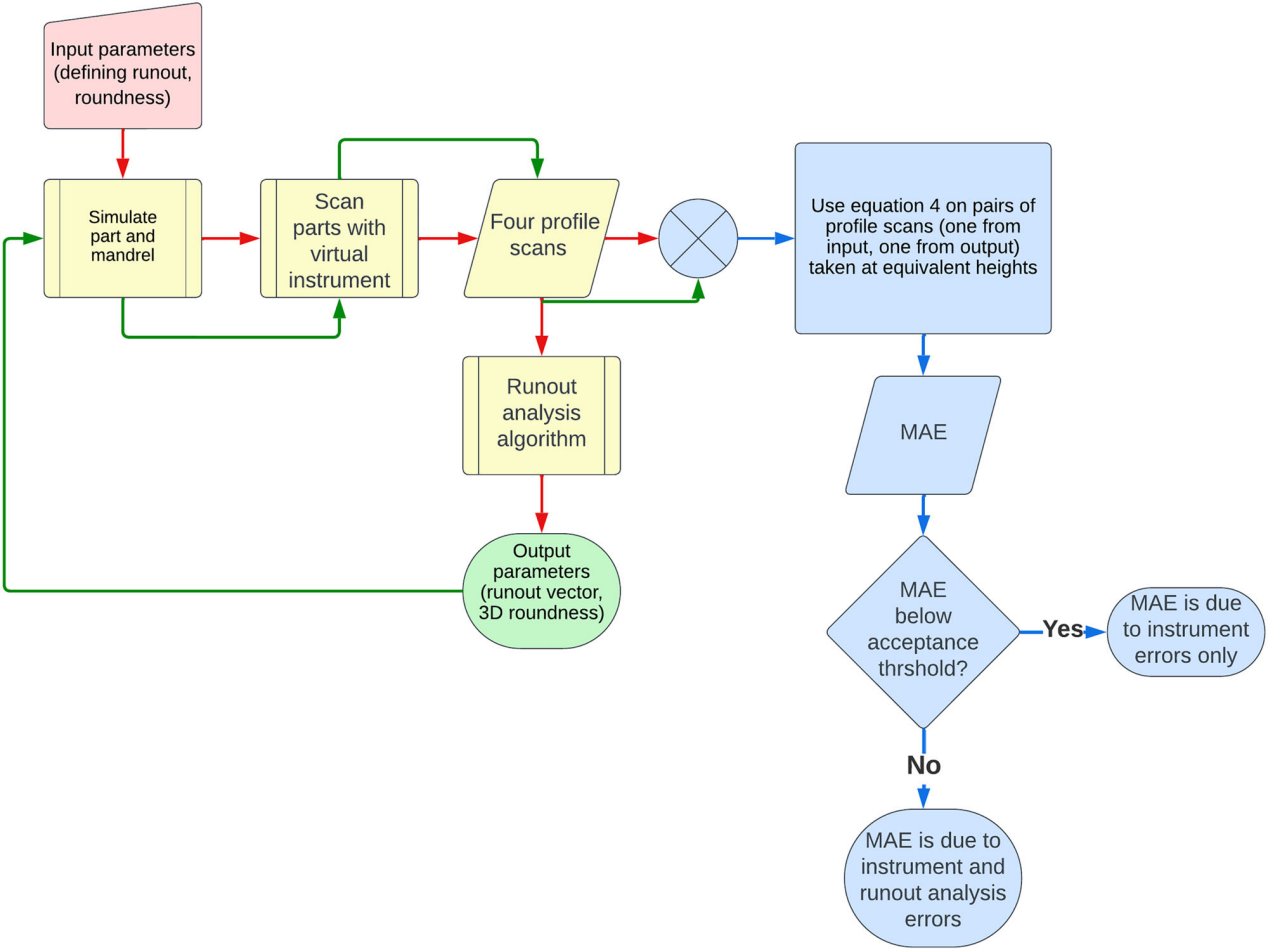
A flow diagram illustration the process by which the mean absolute error (MAE) is calculated for a given set of input parameters. The output of the first cycle (red path) is used as the input for the second cycle (green path). These branches are then combined (blue path) and profile scans taken at equivalent heights are used to calculate the MAE with Eq. (4). To find the contribution to the MAE caused by the instrument, the first cycle is repeated a statistically significant number of times, and an array of MAE values calculated by cross-comparing these. The fluctuation in the MAE is then caused by the system only, and set as the acceptance threshold.

The parts generated from these parameters are considered identical if the virtual instrument produces identical point clouds, when scanning from identical positions. However, there are some sources of inaccuracy, namely: the runout algorithm, and the virtual instrument itself (e.g. surface noise and the quantisation of the simulation). If these sources can be separated out, then this sets the acceptance threshold for whether the two sets of parameters produce identical parts. Any value below the acceptance threshold will be due to the contribution of the instrument, any value above this will give errors caused by the runout analysis. The acceptance threshold is taken as 0.5%, which was computed by generating a set of 500 surfaces with identical defining parameters. Each set was compared to each other, and the maximum variation of the MAE computed. This MAE was set as the acceptance threshold. For clarity, this process has been illustrated in Fig. 3.

In the results presented below, the MAE has been presented consistently. Any time the MAE begins to increase above the acceptance threshold, examples of the profiles scans have also been shown, to visually demonstrate the effects of the 3D runout model failing to characterise the part.

## Results

### Varying individual parameters

**Fig. 4:**
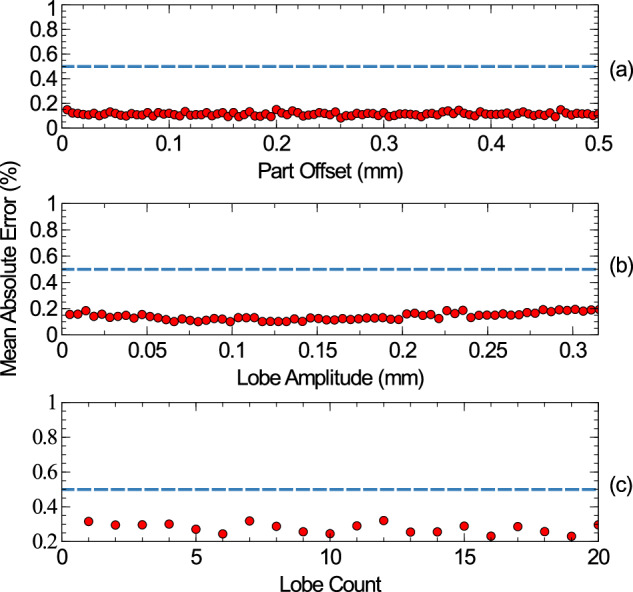
The mean absolute error graph for a set of cylinders with 2*mm* radii, as various runout parameters are varied. There parameters are part offset **a** lobe amplitude **b** and lobe count **c**. In the bottom case, lobe amplitude if fixed at 0.5 *mm*, and in the centre case lobe count is fixed at 2. No other runout parameters are varied. The dashed blue line represents the acceptance threshold, below which all uncertainties are below the magnitude of the errors due to the virtual instrument, above this errors are due to the method of runout analysis. All errors are below the threshold and show no net trend, indicating varying these parameters do not affect the analysis.

Using the MAE-based evaluation method, not all parameters were determined to have an individual effect on the proposed runout algorithm. The features which did not cause any additional inaccuracy as they were varied were: the lobe count, lobe amplitude, and concentricity. The results for these are shown in Fig. 4 below. In all cases, the mandrel had no runout and the part had a surface roughness of *δr* = ± 5*μm* and a radius of 2 mm. When only the lobe count was considered, it was varied between 0 and 19. This could not be fully separated from the lobe amplitude, so this was kept consistent at 0.5 mm. When the lobe amplitude was varied, the lobe count was kept fixed at 2. When only part offset was considered, it was varied between 0 *mm* and 0.5 mm. All other runout parameters of the part were kept at 0 throughout these simulations.

Details of the runout parameters which did affect the runout algorithm are detailed below.

#### Part surface roughness

**Fig. 5:**
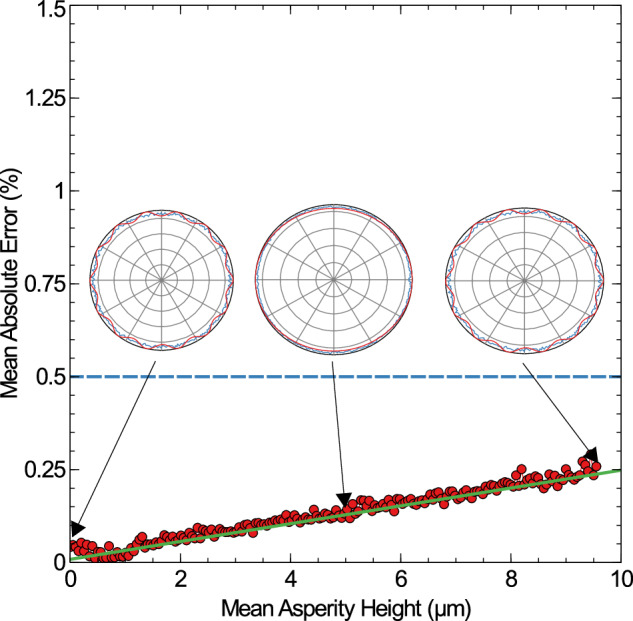
The mean absolute error graph (red) for a set of cylinders with a 2 *mm* radii and rough surfaces. The mean surface roughness increases from 0 *μm* to 10 *μm*. The acceptance threshold is drawn in dashed blue. A linear fit (green) has been made to the data to determine at which roughness values the physical features will no longer be accurately captured, determined as 20 *μm*. Three example surface profiles and the results of the fitting algorithm have been inset, for roughness values of 0, 5, and 9.5 *μm* to illustrate the fits.

As previously mentioned, various machining processes are known to produce different surface roughness values, both in the asperity heights and their distribution. The most common distribution for freshly machined surfaces is Gaussian^29,30,34^, so only this is investigated here, with asperity heights between 0 *μm* and 10 *μm*. This data is shown in Fig. 5 below.

Increasing the mean surface roughness makes the physical features harder to discern, which is to be expected as roughness may be considered as noise on the ideal profiles and therefore increase the likelihood of errors in any fitting algorithm. The main feature that the model fails to capture is the lobed radii in Eq. (3); the higher spread of data in the point cloud allows for a higher range of valid radii. As a consequence, the lobe count is also not accurately captured, although this has no net trend and instead varies randomly with the noise. A linear fit has been made to the calculated MAE, which was used to determine when the fluctuations on the lobing crosses the acceptance threshold, which was determined to occur at a mean surface roughness of 20 *μm*. At this roughness value, the the roughness-induced noise would have a significant enough influence on the profiles that the error on the calculated lobe count/amplitude will be greater than the error caused by the instrument. This means the error will be greater than the quantisation of the instrument, and the input and output surfaces can no longer be considered nominally identical, with the cause of this deviation being the runout analysis method.

#### Part tilt

**Fig. 6:**
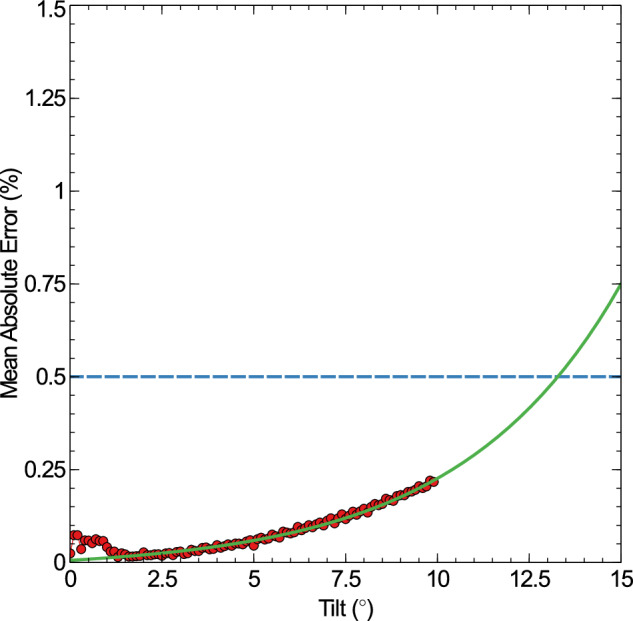
The mean absolute error (MAE) graph (red) for a set of cylinders with a 2*mm* radii and rough surfaces, as a tilt is slowly applied. The mean surface roughness is 5 *μm*, and the tilt varies between 0^∘^ and 9. 9^∘^. As the tilt increases, the surface properties slowly deviate from the expected values. Based on an exponential fit (green), MAE = 0.025e^0.2284*θ*^ − 0.019, the cylinders will pass the acceptance threshold (dashed blue) at a tilt of 13.29^∘^.

The final individual parameter to test is the tilt of the part. A range of tilts between 0° and 9. 9° are tested and applied to the part, the mandrel remains untilted throughout. Larger tilts were not possible, simply because they increased the volume of the simulation beyond the storage capacity of the GPU the data was processed in. This is shown in Fig. 6.

An interesting feature is that the MAE decreases initially as the part is tilted, shown by the slight bump in the graph between tilts of 0° and 1°, this is due to the noise threshold. As there is no lobing of the part, surface noise allows for low amplitude lobing (of any value of *n*) to fit to the parts. Once the part is tilted enough that a defined shape appears above the noise threshold, this no longer occurs and a fixed value of *n* is recovered. The MAE steadily increases as more extreme tilts begin to be applied, however all values remain below the error threshold, up to the maximum value of 9. 9° tested here. An exponential curve of the function *MAE* = 0.025e^0.2284*θ*^ − 0.019 fits well to this curve, and predicts that the tilt angle which brings the MAE above the acceptance threshold is 13.29°. At any tilt greater than this angle, the input and output parameters can no longer be considered comparable. The parameter which fails to be captured in this case is the lobe count/amplitude, as the cross section of a tilted cylinder is an ellipse, and the perturbation of the shape is sufficient that the runout algorithm cannot compensate without additional analysis steps.

### Varying combined parameters

In the previous section, it has been demonstrated how varying one runout parameter, deviating from the ideal cylindrical case, affects how accurately the runout model may recover these parameters. These results can then be used to identify what happens when various parameters are combined, key examples of which are shown here. Once again, the MAE is shown, however this time as a 2D surface map, due to the dimensionality of the parameters involved.

#### Part lobe count and part surface roughness

**Fig. 7:**
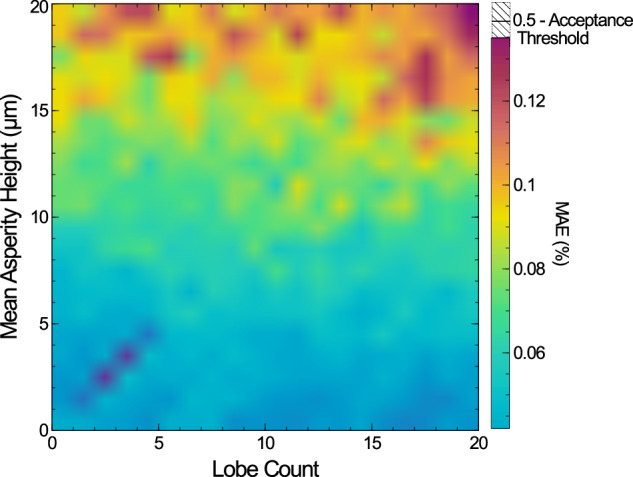
A 2D surface map of the mean absolute error (MAE) of a set of 2 mm radii cylindrical parts, as the lobe count and Gaussian surface roughness are both varied. No net trend in the mean absolute error is observable in the lobe count, and it is instead governed almost solely by the surface roughness.

The first combination analysed is a lobed surface with varying surface roughness. Lobe amplitude of the part is set as 0.5 mm, and the lobe count increases from 0 to 20. The surface roughness is defined by a Gaussian distribution of asperities and increased from 0*μm* to 5*μm*. It must be noted that, from a physical standpoint, there is no well defined difference between low intensity/high amplitude surface lobing and surface roughness. Under these conditions there would be no distinction between the two parameters. However, lobing caused by chatter and ill-mounted parts during various machining processes cause lobing of sufficiently high amplitude compared to the surface roughness that the two parameters may be considered unique enough to analyse. The results of these simulations are in Fig. 7.

There is no observable joint-correlation when the two like-parameters are varied, and lobing has minimal effect on the ability to resolve the input parameters. This was confirmed using a 2D polynomial fit to the data: *f*(*n*, *δr*) = P00 + P01*n* + P10*δr*. The ratio between the coefficients P01:P10 was found to be 1:231, confirming no measurable contribution from the lobe count. Instead the MAE is determined almost solely by the surface roughness and there is no cross-talk between the parameters while they have values reproducible in common machines.

#### Part tilt and mandrel tilt

**Fig. 8:**
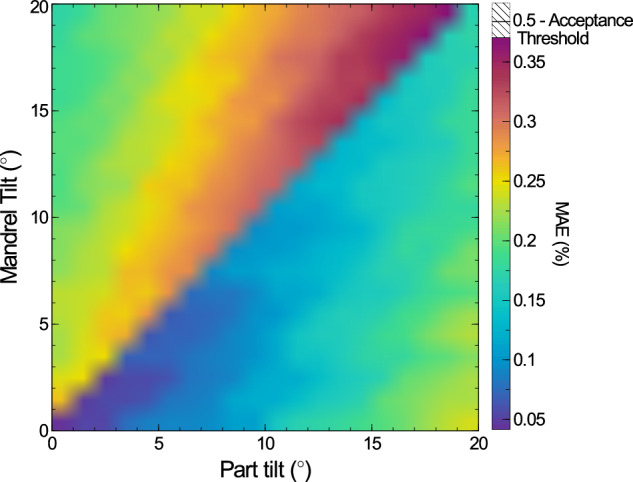
A 2D surface map of the mean absolute error (MAE) of a set of 2 mm radii cylindrical parts, as the tilt of the part and the mandrel it is mounted on are increased from 0^∘^ to 3. 8^∘^. The two tilts are aligned 45^∘^ from each other. A step in the mean absolute error can be seen when the two tilt magnitudes are equal.

The next set of features tested were a combination of tilts, applied to both the part and mandrel. The magnitudes of the tilts both ranged between 0° and 3. 8°, however they were applied along different directions. The part tilt was applied purely along the $\hat{x}$ axis, and the mandrel tilt had equal magnitude along both the $\hat{x}$ and $\hat{y}$ axis. In this way, the two tilt magnitudes are not fully separable, and this is therefore the most difficult combination to measure. The results of this analysis are shown in Fig. 8. The range of tilt angles tested has been reduced down from 9. 9°, used in the single parameter simulations, to reduce the computation time required.

An important feature to note is that the MAE increases when the mandrel tilt is greater than the part tilt, as the cross section of the part is no longer well defined by Eq. (1), instead being a superposition of two offset cylinders of equal magnitude. As the tilt of the mandrel increases and is no longer comparable to that of the part, and is therefore easier too decouple, the MAE actually begins to decrease again, although it is still greater than if the mandrel tilt was minimised as it is still partially capturing the mandrel tilt. That is to say, the error on this runout method is maximised when the part and mandrel have comparable amounts of tilt. Minimising mandrel tilt is therefore important, although has little impact as long as it is smaller than the tilt of the part.

#### Part lobe count and part tilt

**Fig. 9:**
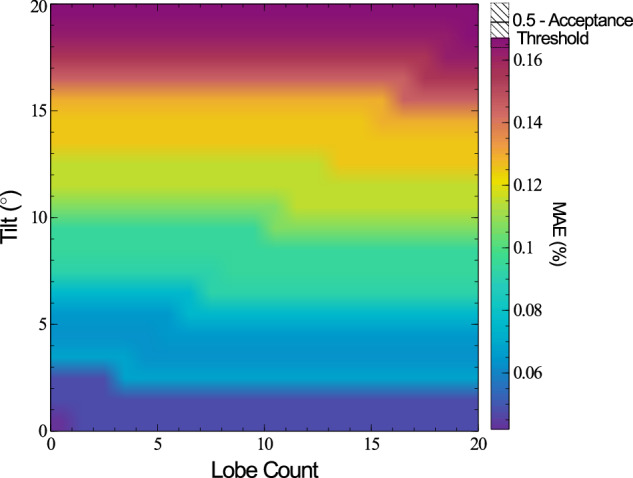
A 2D surface map of the mean absolute error (MAE) of a set of 2 mm radii cylindrical parts, as the part tilt and lobe count are both increased. The mean absolute error is dependant on both parameters, however the tilt is the main factor.

The final combination of features investigated here is part lobing and tilt. These results are shown in Fig. 9. As before, reduced down from 9. 9° to 3. 8°.

Once again, the key determinate feature in capturing the runout properties is the tilt of the part, with almost no contribution from the lobing, indicating a robust method of capturing these properties. Based on the same exponential fitting method used in Fig. 6, increasing the lobe count from 0 to 20 will cause the MAE to pass the acceptance threshold at 9.96° instead of 13.29°, effectively identical for practical applications.

### Measurement conditions summary

The overall measurement protocol of the proposed method may then be summarised as follows.

Four profile scans are required for the measurement process: one at the top and bottom of the part measurement zone, and one at the top and bottom of the fixturing. A runout vector should then be produced for the fixturing, and combined assembly. Removing the fixturing vector from the assembly vector gives the total runout vector across the measurement zone of the component. During measurement, the measurement conditions must meet the following criteria:

- A part tilt less than 13°, relative to the sensor
- Adequate fixturing such that mandrel tilt is less than the part tilt
- Surface roughness needs to be no greater than 20 *μm* larger than the feature sizes

Failure to meet these conditions causes the measurement to no longer be conformant to the measurand, resulting in an inaccuracy in the analytical results. Some preliminary experimental data, sampled under these conditions, are also shown in the Supplementary Material (Item 2).

## Conclusion

Overall, a method of quickly characterising the three-dimensional runout of rotationally symmetric components has been defined, based on taking two profile measurements and computing a runout vector. A method of differentiating this runout vector from that of any mounting was also defined. To increase the accuracy of this runout vector, a method of measuring 3D roundness was developed. This was an expansion of older least-squares circle methods, using a formula for a lobed cylinder.

The limitations of the proposed method were investigated through a virtual instrument and assessed with a novel approach for evaluating errors derived from these simulations. Through this process, it was established that the key features which prevent the accurate capture of the runout parameters are the tilt of the component and the surface roughness. These key parameters were then investigated in combination, with the main additional limitation observed being that the runout of any mounting must be lower than that of the component that is mounted on it. The scale of these limiting properties are sufficiently large that they can be measured in real systems and not obstruct runout measurement in a practical setting.

This proposed method has been designed in such a way that it is extensible, allowing for features such as a moving sensor to account for tilt, without changing the underlying modelling and definitions. The accuracy of the model from minimal data, while being able to adapt it as further needs arise, makes this a viable method to employ in real world situations. The work presented here is purely based on a virtual setup, and the work to integrate this method into a real system is currently ongoing and will be presented in a separate publication soon.

The model itself is also not limited to the linear cases modelled here, and can be extended to accounted for curved surfaces, much in the same way that the definition of polygonal meshes to quantise an object is used in computer aided design and finite element modelling scenarios. This method could be extended to higher fidelity as hardware and computational methods advance, allowing the basic method to be retained permanently, even on more complex and non-linear geometries as computational analysis methods advance. Analysing a rate of change of runout in this case would be ideal when components need to be fitted together, but pose an interesting challenge for part selection. Integrating machine learning methods, trained via our MAE method, into the runout measurement process could provide the necessary computational intelligence required to improve the accuracy and speed of our runout method to reach such levels of smart optimisation.

## Supplementary information

Transparent Peer Review file

Three-Dimensional Runout Characterisation For Rotationally Symmetric Components - Supplementary Information

## Data Availability

All data generated and analysed are either presented in this publication or will be made available on request to the corresponding author.
