# Supplementary material for: Three-dimensional runout characterisation for rotationally symmetric components
Source: Commun Eng. 2025 Feb 12;4:19. doi: 10.1038/s44172-025-00354-0 (PMC11821993; doi:10.1038/s44172-025-00354-0)
Supplement: Supplementary file 2 — Three-Dimensional Runout Characterisation For Rotationally Symmetric Components - Supplementary Information [file 44172_2025_354_MOESM2_ESM.pdf]

Item 1: Development Pipeline

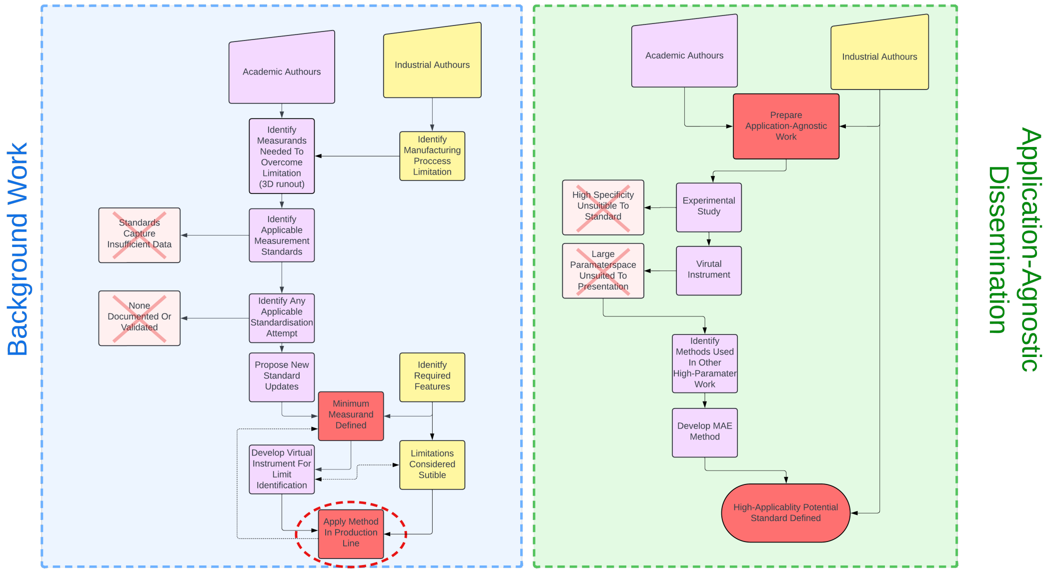

Figure 1: The development pipleline that led to the work outlined in this manuscript. The initial background workflow was a commercially-sensitive collaboration between the academic and industrial authors. This was streamlined and developed into the application-agnostic work presented here. One element, circled in red, was not replicated during the transfer. This was a high-specificity case study and deemed unsuitable to present within the application-agnostic framework of a broad-scope measurement technique. The authors intend to present a detailed case-study in a separate publication, focused on analysis and application within a specific sub-field.

Item 2: Experimental Sample Data

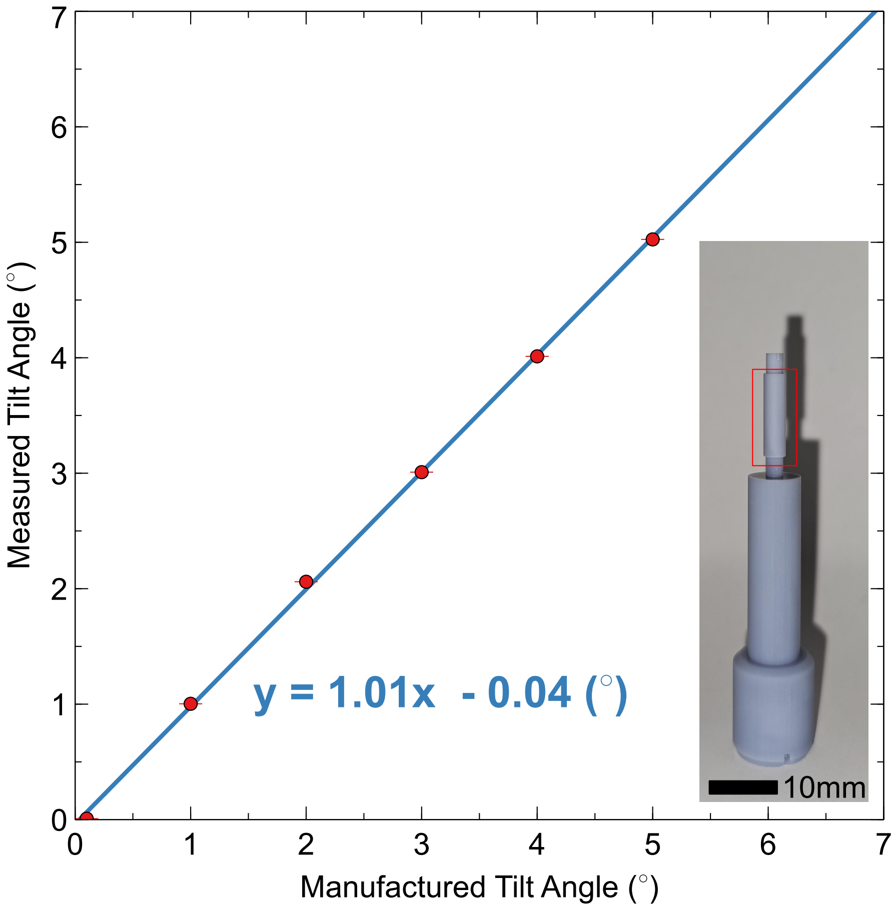

Figure 2: Example experimental data of a validation artefact measured with the method outlined in this work. Four measurements are taken along the artefact, and post-processed with the 3D runout method. Compared are the manufactured angles of 5 artefacts, and the angle recovered using the 3D runout method, showing good correlation. Inset is an image of the validation artefact, with scale bar. The region within the red bounding box is a cylinder, manufactured with a known tilt angle, the "part". The remainder is the "mandrel", with the four measurement zones selected using the method outlines in this work. Remaining features are registration marks.
