# Supplementary material for: Three-dimensional runout characterisation for rotationally symmetric components
Source: Commun Eng. 2025 Feb 12;4:19. doi: 10.1038/s44172-025-00354-0 (PMC11821993; doi:10.1038/s44172-025-00354-0)
Supplement: Supplementary file 1 — Transparent Peer Review file [file 44172_2025_354_MOESM1_ESM.pdf]

This file contains all reviewer reports in order by version, followed by all author rebuttals in order by version.

nature portfolio

Peer Review File

Three-Dimensional Runout Characterisation For Rotationally Symmetric Components

Corresponding Author: Dr Christopher Tompkins

Version 0:

Reviewer comments:

Reviewer #1

(Remarks to the Author)
This paper presents a method of characterising the runout of rotationally symmetric machined parts. The topic is interesting in engineering and practical technology. However, the theoretical depth and paper workload are limited in somewhat, the experiment setup and procedure description is quite not enough.
Suggestions and Comments:
(1) The theoretical model is considered as a little simple idealized one.
(2) The experiment setup and procedure description should be given in detail.
(3) The method comparison and verification procedure are not sufficient.

Reviewer #2

(Remarks to the Author)
1. Overview and General Recommendation
The paper presents a novel approach for the three-dimensional characterization of runout in rotationally symmetric components, employing a method that combines virtual instrument simulation with an innovative evaluation technique for simulation errors. This research offers some potential for improving component quality and manufacturing efficiency.
The methodological innovation and the potential applicability of the research in industrial settings are particularly noteworthy. The authors have successfully demonstrated the method's efficacy using a comprehensive virtual instrument simulation, though the paper would benefit from additional clarification on transitioning this method from a simulated to a practical environment.
Despite its strengths, the manuscript requires improvements in several areas to enhance clarity, structure, and the depth of its theoretical and practical contributions.Although this article has minor problem, the publication of this paper is recommended.

2. Minor Comments
1) Clarity and Structure of the Manuscript
Research Framework: The paper would benefit from a clearer presentation of the research framework. A flowchart illustrating the research ideas, the development of the virtual instrument, and the step-by-step process of the 3D runout characterization would significantly enhance reader understanding.
Methodological Details: While the virtual instrument and the novel evaluation technique are innovative, the description of these methods feels somewhat cumbersome. Simplifying these descriptions and focusing on the core principles and advantages of the method could make the paper more accessible to a broader audience.
2) Literature Review and Theoretical Contribution
Combining relevant literature with existing methods: A more detailed comparison with current 2D and 3D measurement techniques will help to understand the advantages and potential limitations of the proposed methods.
Literature Review: The introduction should be expanded to include a more comprehensive review of existing methods for runout characterization and their limitations. This would provide a stronger justification for the proposed method and highlight its novelty more effectively.
Theoretical implications: The paper should discuss the theoretical implications of the proposed method more thoroughly. Specifically, how does the method contribute to existing knowledge? What is the theoretical basis for the difference from existing methods?
3) Practical Applicability and Case Study
Real-world Application: The transition from virtual to real-world applications is a critical aspect that seems underexplored. Including a section on potential challenges and strategies for implementing the proposed method in industrial settings would be highly beneficial.

Case Study: While the virtual instrument and simulation provide a strong foundation, additional validation using real-world measurements would significantly enhance the credibility and applicability of the findings. If possible, a case study demonstrating the application of the method in a real manufacturing environment would significantly strengthen the paper. This could provide insights into the method's practical challenges, limitations, and benefits.
4) Visual Presentation
Figures and Tables: The format and style of figures and tables should be unified to maintain consistency throughout the paper. Additionally, optimizing the layout of these visual elements could improve readability and the overall presentation quality.
5) Future Work
Extension of the Method: The discussion on future work should elaborate on potential extensions of the proposed method. This might include adapting the method for different types of components, integrating it with other manufacturing processes, or enhancing it with machine learning techniques for improved accuracy and efficiency.

Reviewer #3

(Remarks to the Author)
The paper presents a novel method that accurately characterizes the three-dimensional runout of rotationally symmetric components using minimal surface information. Additionally, a new technique for assessing simulated errors in virtual instrument measurements is developed. Overall, the quality of the manuscript needs to be substantially improved, and the contributions are not strong enough. Major concerns are as follows:

1.The paper fails to adequately explain the practical background and value of the research, lacking a thorough explanation of why there is a need for rapidly characterizing the three-dimensional runout of cylindrical components and its potential applications in engineering or manufacturing fields.

2.Although a new method for defining and measuring three-dimensional runout is proposed, the paper lacks direct comparisons with existing methods, failing to demonstrate its innovativeness. No mention is made of similar studies, nor is the method's superiority over existing ones explicitly stated.

3.While virtual instruments are used for simulated experiments in the paper, no comparisons are made with existing methods or standard measurement techniques. There is a lack of sufficient experimental data to assess the performance and accuracy of the new method.

4.The interpretation of experimental results is abstract, lacking specific data support and quantitative analysis. In discussing the impact of key parameters on the results, insufficient experimental data and analysis are provided, leading to a lack of credibility and comprehensibility in the conclusions.

Version 1:

Reviewer comments:

Reviewer #1

(Remarks to the Author)
An experiment setup structure principle and photo should be provided at least.

Reviewer #2

(Remarks to the Author)
Overall, the modifications have addressed many of the initial concerns. The inclusion of a workflow diagram and other efforts significantly enhance the manuscript's readability and comprehensiveness. The innovative approach to three-dimensional runout characterization using virtual instruments is well-presented. But some minor issues can be further modified.
1) The new "method summary" section is a valuable addition. However, it could be further refined to succinctly highlight the method's key steps and advantages. Besides, why the development pipeline was provided in the appendix, instead of in Section 2? What’s more, where is A.2 if the Appendix includes A.1?
2)Providing examples of potential applications and their theoretical underpinnings would help illustrate the method's broader impact.
3)Detailed explanation of how the proposed method compares to existing methods in terms of accuracy, reliability, and applicability would be appreciated.

Reviewer #3

(Remarks to the Author)
Issue 1:
Reviewer Comment: The paper fails to adequately explain the practical background and value of the research, lacking a

thorough explanation of why there is a need for rapidly characterizing the three-dimensional runout of cylindrical components and its potential applications in engineering or manufacturing fields.

Authors' Response: The authors explained that the method applies to various parts, from gears to bearings and pistons, and they aimed to avoid high detail that might limit the work to specific fields. They corrected the term "cylindrical" to "rotationally symmetric" components and included more background information on current measurement standards and their limitations.

Evaluation: The authors provided a detailed explanation of their decision to keep the application general and added broader background information. These modifications significantly improved the explanation of the study's background. I agree with the authors that high-detail applications can be published in subsequent specific studies. Therefore, I am satisfied with the changes made to this section.

Issue 2:
Reviewer Comment: Although a new method for defining and measuring three-dimensional runout is proposed, the paper lacks direct comparisons with existing methods, failing to demonstrate its innovativeness. No mention is made of similar studies, nor is the method's superiority over existing ones explicitly stated.

Authors' Response: The authors acknowledged this point and explained that no existing standardized methods can gather spatial information about the runout of parts, which led to the development of their method. They added a new workflow diagram to illustrate their development process.

Evaluation: The authors' response and the new workflow diagram effectively explain the uniqueness of their method and its development background. These additions successfully demonstrate the method's innovativeness and provide the necessary context. I find this response and the modifications satisfactory.

Issue 3:
Reviewer Comment: While virtual instruments are used for simulated experiments in the paper, no comparisons are made with existing methods or standard measurement techniques. There is a lack of sufficient experimental data to assess the performance and accuracy of the new method.

Authors' Response: The authors explained that their method was developed alongside a commercially sensitive case study, which they plan to publish separately. They provided further clarification and plan to detail the case study in subsequent application-specific research.

Evaluation: The authors' explanation is reasonable and clearly outlines why they did not include specific case studies. Their plan to detail the practical applications in future studies is appropriate. However, the current manuscript still lacks experimental data and comparative analysis, which are crucial for assessing the new method's performance. I suggest adding some key experimental data and comparisons in the current manuscript to strengthen its credibility and completeness.

Issue 4:
Reviewer Comment: The interpretation of experimental results is abstract, lacking specific data support and quantitative analysis. In discussing the impact of key parameters on the results, insufficient experimental data and analysis are provided, leading to a lack of credibility and comprehensibility in the conclusions.

Authors' Response: The authors explained the difficulty in presenting a five-dimensional parameter space and referenced other engineering and AI works using large parameter spaces. They provided a more detailed description of the parameter space and mentioned they could add complete datasets to an open repository.

Evaluation: The authors' response and modifications improved the explanation of the parameter space and provided a clearer analysis method. However, to enhance the paper's credibility, I suggest including more specific experimental data and graphs in the main text, even if linked to an appendix or online repository, to significantly improve the paper's credibility and comprehensibility.

Open Access This Peer Review File is licensed under a Creative Commons Attribution 4.0 International License, which permits use, sharing, adaptation, distribution and reproduction in any medium or format, as long as you give appropriate credit to the original author(s) and the source, provide a link to the Creative Commons license, and indicate if changes were made.
In cases where reviewers are anonymous, credit should be given to 'Anonymous Referee' and the source.
The images or other third party material in this Peer Review File are included in the article's Creative Commons license, unless indicated otherwise in a credit line to the material. If material is not included in the article's Creative Commons license and your intended use is not permitted by statutory regulation or exceeds the permitted use, you will need to obtain permission directly from the copyright holder.
To view a copy of this license, visit https://creativecommons.org/licenses/by/4.0/

Reviewer Replies to "Three-Dimensional Runout Characterisation For Rotationally Symmetric Components" V1

June 18, 2024

1 Reviewer 1

This paper presents a method of characterising the runout of rotationally symmetric machined parts. The topic is interesting in engineering and practical technology. However, the theoretical depth and paper workload are limited in somewhat, the experiment setup and procedure description is quite not enough.

We thank the reviewer for their comments; we have taken them into account, and in the next sections we try to highlight the engineering and practical-sciences applications of our work in more detail. We hope the changes and clarifications we have made, in response, are satisfactory.

1.1 *The theoretical model is considered as a little simple idealized one.*

The reviewer raises an interesting question: are the parameters that we model sufficient? In general it is assumed that machined parts have an idealised shape, and that flaws in the machining process introduce a group of defects to the part, grouped by the current measurement standards (such as ISO 12181-1:2011 and ISO 1101-2017) into radial and axial runout, roundness deviation, and roughness. Since the standards do not consider any other physical properties, we decided to look at previous experimental work (such as [1–4]) which quantify these features, and build a virtual instrument that includes all these parameters. As this work was done in collaboration with a manufacturing company, we also reviewed with them the suitability of these parameters, and if more were needed. Of course, even if only these parameters are considered at this stage, in future other parameters could also be added, if needed. This aspect has been also clarified

in the paper. In particular, in the background of this work, we have expanded the introductory section to further clarify the current standards. We have also briefly explained it again when introducing the model.

The current 1D standards of roundness measurement have remained effectively unchanged since their implementation in 1985 [11], before current digital analysis methods became widespread or affordable. Their limitations were transferred over from by-hand methods, and have been maintained for ease of use and traceability. However, technological advancements within the current automation and industry 4.0 framework mean that extending these standard beyond simple magnitude measurements, maintaining backwards compatibility and using the already increasing computation of smart manufacturing, has now become both feasible and desirable. An addition of more physically representative information in the measurement, kept concise, would provide more knowledge of the measurand and allow for techniques like smart pairing of components using available machinery, instead of resource-heavy retooling/rejection. Outlined in this paper

is, to the authors knowledge, the first proposed extension to these standards: a fully backwards-compatible extension which characterises runout in 3D, as a single-valued runout vector. This new method is tested and characterised to, determine the limits of the physical features it can measure, using another modern manufacturing tool: a virtual instrument.

3 The proposed 3D runout model

The proposed model builds on simple magnitude-only measurement standards, to a more comprehensive 3D method which provides spatial-geometric information. Only the overall runout of the part is considered in this work, measuring from one end of the part to the other, to determine the net runout across the length of a parts fixturing. This runout model extends the standard definition of runout [7] by giving it magnitude and direction – a runout vector. This vector is defined in cylindrical polar coordinates, giving a magnitude in the $\hat{r}$

1.2 *The experiment setup and procedure description should be given in detail.*

This paper focuses not on a specific experimental work, but instead aims to develop a novel measurement pipeline that could be used for different applications. In this respect we can affirm that we are presenting a new method in its discovery phase. For this purpose, we have left it application-agnostic for the sake of broad applicability. In order to show the broad application of the proposed method, we have introduced and validated it by using a virtual instrument. We have added two clarifications to the manuscript to explain this: the following additions to the text, and the following workflow diagram in the appendix to explain the development process. The experimental study we show in this diagram remains unexplained due to the sensitivity of it to our industrial authors. We instead plan to replicate a high-specificity case-study in a work dedicated solely to this, with less applicability to engineering/STEM as a whole but of more interest to the specific sub-field.

4 Modelling virtual parts

It has long been been established that, within the physical constrains of non-elastic and non-environmentally aged surfaces, there are a small number of parameters which define their shape. These parameters are commonly grouped into surface roughness and surface profile parameters (further broken down into runout and roundness). Since these physical features are those that cause runout and roundness deviations, it is these that have been aggregated to form the instrument, and that it aims to measure with the proposed 3D runout method. To keep applicability broad, no component-type specific (e.g. gear) features are considered, instead the components are kept as general cylinders with runout and roundness perturbations added to their shape. These perturbations cover the complete set of runout, roundness, and surface roughness features expected on physical parts from previous experimental work and known measuremnt protocols [3, 10, 23–27].

The virtual components themselves were defined on-grid; a 3D byte array was [illegible]

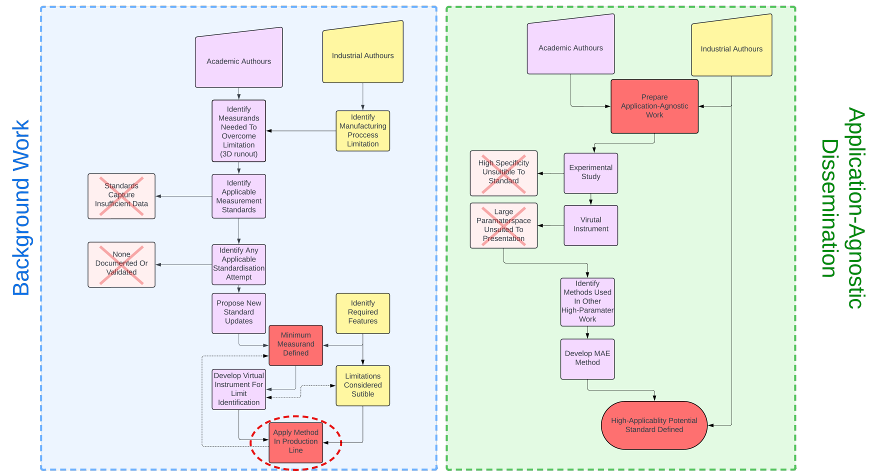

Figure A.1: The development pipleline that led to the work outlined in this manuscript. The initial "background" workflow was a commercially-sensitive collaboration between the academic and industrial authors. This was streamlined and developed into the application-agnostic work presented here. One element, circled in red, was not replicated during the transfer. This was a high-specificity case study and deemed unsuitable to present within the application-agnostic framework of a broad-scope measurement technique. The authors intend to present a detailed case-study in a separate publication, focused on analysis and application within a specific sub-field.

1.3 *The method comparison and verification procedure are not sufficient.*

The proposed method includes the evaluation of a five-dimensional parameter space, and of course when it comes to represent this in two-dimensions, there are some unavoidable challenges. Other AI-enhanced engineering papers face the same challenges (see for example works from [5–8]. In these methods, conformance is measured by comparing deviation between input and output datasets. A threshold needs to be defined in order to assess the results, as random fluctuations due to specific characteristics, such as roughness, will mean equivalent surfaces are not necessarily identical. In our case, this threshold was determined as 0.5%. For this reason, for a given parameter, we have consider the behaviour of the MAE and assessed whether conformance was maintained when a parameter is altered. For example, in figure 5 of the manuscript, we have shown what happens when increasing part tilt by tracking whether the MAE increases beyond the acceptance threshold. Of course, all raw data will be made available in the data repository and can be prepared into hundreds of additional

graphs the readers may find helpful. We have added the following clarifications to the text, to include other works that have followed the same process.

ment accuracy. In this case, features may be a combination of part and mandrel parameters, such as a tilted part on a tilted mandrel .

As there is a five-dimensional data-space, the conformance of each individual parameter cannot be directly plotted in any concise manner [17–20]. For this reason the mean absolute error (MAE) of the residuals between input (I) and output (O) datasets are calculated following works by [17–20]:

$$MAE = \frac{\sum_i^n |I_i - O_i|}{n\bar{I}}, \tag{4}$$

where i is each element within the set. In this case, the datasets are profiles scans of a generated surface with the parameters: tilt, part offset, lobe amplitude, lobe count, and mean asperity height. Two profiles are therefore not identical unless all parameters which define them are identical. To generate the two sets of profiles, 3D part-mandrel assemblies are generated and analysed with the virtual instrument, one with the input parameters (I), and one with the output parameters (O). The input parameters are user defined, and the output parameters were obtained by scanning the initial part and then recovering the runout/roundess parameters with the proposed 3D runout model, then using these to generate a new part. This new part is then measured, to generate new

9

profile scans for comparison.

References

1. Yu, N. & Polycarpou, A. A. Contact of Rough Surfaces With Asymmetric Distribution of Asperity Heights. *Journal of Tribology* **124,** 367–376. ISSN: 0742-4787 (July 2001).
2. Molnar, V. Asymmetric Height Distribution of Surfaces Machined by Hard Turning and Grinding. *Symmetry* **14.** ISSN: 2073-8994 (2022).
3. Elerian, F. & Abou Eleaz, M. Methods of Roundness Measurement: An Experimental Comparative Study (Aug. 2021).
4. Yu, N. & Polycarpou, A. Combining and Contacting of Two Rough Surfaces with Asymmetric Distribution of Asperity Heights. *Journal of Tribology-transactions of The Asme - J TRIBOL-TRANS ASME* **126** (Apr. 2004).
5. Qi, J., Du, J., Siniscalchi, S. M., Ma, X. & Lee, C.-H. On Mean Absolute Error for Deep Neural Network Based Vector-to-Vector Regression. *IEEE Signal Processing Letters* **27,** 1485–1489. ISSN: 1558-2361 (2020).
6. Error Metrics and Performance Fitness Indicators for Artificial Intelligence and Machine Learning in Engineering and Sciences. eng. *Architecture, Structures and Construction* **3,** 499–517. ISSN: 2730-9886 (2023).
7. Wang, Z., Bovik, A., Sheikh, H. & Simoncelli, E. Image quality assessment: from error visibility to structural similarity. *IEEE Transactions on Image Processing* **13,** 600–612 (2004).
8. Yuan, S., He, Z., Zhao, J., Yang, Z. & Yuan, Z. Long-term electrocardiogram signal quality assessment pipeline based on a frequency-adaptive mean absolute deviation curve. eng. *Applied intelligence (Dordrecht, Netherlands)* **53,** 20418–20440. ISSN: 0924-669X (2023).

2 Reviewer 2

Overview and General Recommendation The paper presents a novel approach for the three-dimensional characterisation of runout in rotationally symmetric components, employing a method that combines virtual instrument simulation with an innovative evaluation technique for simulation errors. This research offers some potential for improving component quality and manufacturing efficiency. The methodological innovation and the potential applicability of the research in industrial settings are particularly noteworthy. The authors have successfully demonstrated the method's efficacy using a comprehensive virtual instrument simulation, though the paper would benefit from additional clarification on transitioning this method from a simulated to a practical environmentDespite its strengths, the manuscript requires improvements in several areas to enhance clarity, structure, and the depth of its theoretical and practical contributions. Although this article has minor problem, the publication of this paper is recommended.

We thank the reviewer for taking the time to provide may insightful comments on our methods and are glad that they appreciate the comprehensiveness of our analysis, as well as the advantages of virtual instrumentation in difficult scenarios such as this. We have found the reviewers comments have helped us identify some weaknesses in our work, and have restructured it in such a way that we can maximise our dissemination and approachability based on them. The reviewer will find a breakdown of these key changes below.

2.1 *Clarity and Structure of the Manuscript Research Framework: The paper would benefit from a clearer presentation of the research framework. A flowchart illustrating the research ideas, the development of the virtual instrument, and the step-by-step process of the 3D runout characterisation would significantly enhance reader understanding. Methodological Details: While the virtual instrument and the novel evaluation technique are innovative, the description of these methods feels somewhat cumbersome. Simplifying these descriptions and focusing on the core principles and advantages of the method could make the paper more accessible to a broader audience.*

We appreciate the suggestions the reviewer has had on these points. We have taken the following set of suggestions and incorporated them into out work by adding the following three major changes:

1 A workflow diagram to show both the development process, and how the work was transferred into a paper, has been provided in the appendix.

2 A new ”method summary” section, separate form the detailed sub-steps, has been added. This includes more details about the method description.

3 A final summary of our suggested protocol, which summarises the key results and runout methodology, has been included.

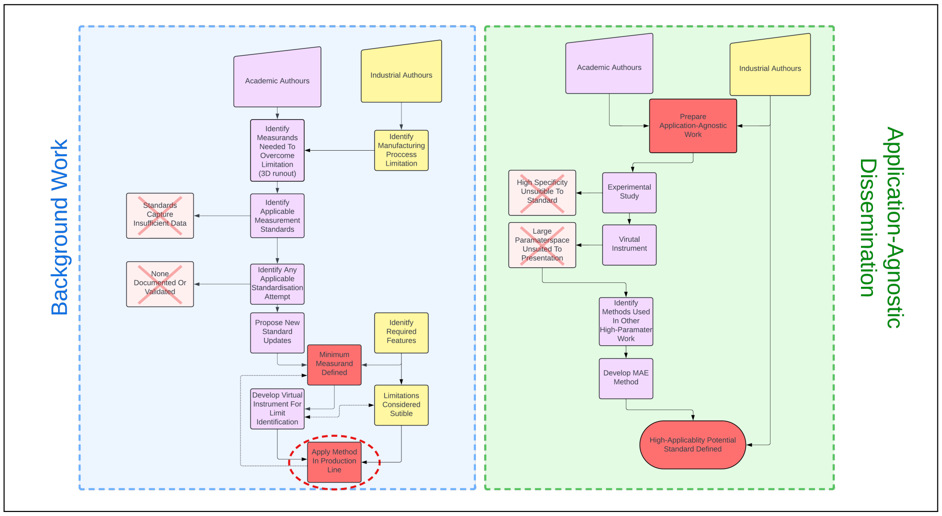

2 Method Summary

The work presented below is a summary of an academic-industrial collaboration to develop a new method of runout analysis for rotationally-symmetric objects. This method contains additional spatial and geometric information of the measurand, directly linking the measurement to its physical properties, to allow for a more physically-representative measurement. The full development pipeline of the work is presented in Appendix A.1.

The first methodology section in this work details the new proposed 3D runout method, listing the measurement process and all relevant measurement quantities. Following the 3D runout definition, a virtual instrument is presented.

The virtual instrument was developed by identifying the relevant physical quantities in the current runout and roundness standards [8, 9], namely: surface lobing, surface roughness, axial runout, and concentricity. Bulk experimental data are then aggregated and developed into a generic rotationally-symmetric part, where relevant parameters are varied based on previous data to iterate over different parts.

The analysis process for these virtual parts is then detailed, using a process similar to that in previous works, where large experimental datasets are analysed [17–20]. In this proposed analysis process, a component generated from the mea-

sured runout parameters is compared to the initial measurand, and conformance between the two is evaluated. If the 3D runout method accurately captures the component characteristics (within some quantified uncertainty in the instrument), then the method will replicate the surface and the two components will be functionally identical.

The conformance in then analysed for a variety of parameters and the limiting properties of the measurement technique identified. These are then summarised, with a recommended measurement protocol detailed afterwards.

6.3 Measurement Conditions Summary

The overall measurement protocol of the proposed method may then be summarised as follows.

Four profile scans are required for the measurement process: one at the top and bottom of the part measurement zone, and one at the top and bottom of the fixturing. A runout vector should then be produced for the fixturing, and combined assembly. Removing the fixturing vector from the assembly vector gives the total runout vector across the measurement zone of the component. During measurement, the measurement conditions must meet the following criteria:

- A part tilt less than 13°, relative to the sensor
- Adequate fixturing such that mandrel tilt is less than the part tilt

- Surface roughness needs to be no greater than $20\mu m$ larger than the feature sizes

Failure to meet these conditions causes the measurement to no longer be conformant to the measurand, resulting in an inaccuracy in the analytical results.

2.2 *Literature Review: The introduction should be expanded to include a more comprehensive review of existing methods for runout characterisation and their limitations. This would provide a stronger justification for the proposed method and highlight its novelty more effectively.*

We have included some additional details in the main body of the text to explain the current measurement methods, and the unfortunate lack of 2D/3D measurement standards. We have highlighted that the current methods only give 1D (magnitude) measurements, which do not contain any spatial information about the part that is measured, and how this limits their usage. These changes are as follows:

The current 1D standards of roundness measurement have remained effectively unchanged since their implementation in 1985 [11], before current digital analysis methods became widespread or affordable. Their limitations were transferred over from by-hand methods, and have been maintained for ease of use and traceability. However, technological advancements within the current automation and industry 4.0 framework mean that extending these standard beyond simple magnitude measurements, maintaining backwards compatibility and using the already increasing computation of smart manufacturing, has now become both feasible and desirable. An addition of more physically representative information in the measurement, kept concise, would provide more knowledge of the measurand and allow for techniques like smart pairing of components using available machinery, instead of resource-heavy retooling/rejection. Outlined in this paper

is, to the authors knowledge, the first proposed extension to these standards: a fully backwards-compatible extension which characterises runout in 3D, as a single-valued runout vector. This new method is tested and characterised to, determine the limits of the physical features it can measure, using another modern manufacturing tool: a virtual instrument.

We now also refer back to this point when introducing the model in detail, to briefly remind the reader what we are building upon:

3 The proposed 3D runout model

The proposed model builds on simple magnitude-only measurement standards, to a more comprehensive 3D method which provides spatial-geometric information. Only the overall runout of the part is considered in this work, measuring from one end of the part to the other, to determine the net runout across the length of a parts fixturing. This runout model extends the standard definition of runout [7] by giving it magnitude and direction – a runout vector. This vector is defined in cylindrical polar coordinates, giving a magnitude in the $\hat{r}$

2.3 *Theoretical implications: The paper should discuss the theoretical implications of the proposed method more thoroughly. Specifically, how does the method contribute to existing knowledge? What is the theoretical basis for the difference from existing methods?*

The reviewer has made some good suggestions, and we hope they do not mind us including them directly. Our method is intended to be a fully backwards-compatible extension to the current measurement protocols, extending them from a simple magnitude-only measurement to a fully 3D measurement. This extra spatial information, which we gain from the minimal-most extra analysis possible, does indeed provide extra context and open the potential for a large amount of knowledge-gain in many fields. We hope that the extra detail we have added into the above literature review, based on the reviewers suggestion, covers this is enough detail.

2.4 *Case Study: While the virtual instrument and simulation provide a strong foundation, additional validation using real-world measurements would significantly enhance the credibility and applicability of the findings. If possible, a case study demonstrating the application of the method in a real manufacturing environment would significantly strengthen the paper. This could provide insights into the method's practical challenges, limitations, and benefits.*

We are in full agreement that an experimental case study to demonstrate our method is valuable, and lead authors are currently in the preliminary stages of preparing one such high-specificity study, with our industrial coauthors. However, the reason we did not include any study such as this was a conscious choice: the method we are proposing has the potential to become a new measurement standard. As the reviewer will be familiar, the development process of these is quite specific. We targeted this work as the initial "method discovery" phase, hoping it to be applicable to engineering/STEM as a whole, and purposefully kept

the scope broad to enhance uptake. We are then hoping to complete a second publication which is a detailed and stand-alone case study, much more targeted to an individual sub-field. As the reviewer will see in the flow diagram we included, thanks to their advice, some commercially sensitive experimental work has already been completed. We have made the following changes to make these choices clear:

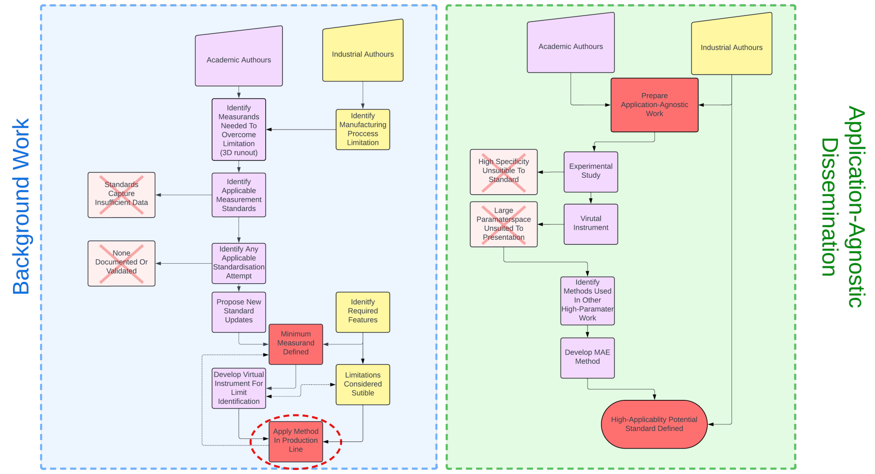

Figure A.1: The development pipleline that led to the work outlined in this manuscript. The initial ”background” workflow was a commercially-sensitive collaboration between the academic and industrial authors. This was streamlined and developed into the application-agnostic work presented here. One element, circled in red, was not replicated during the transfer. This was a high-specificity case study and deemed unsuitable to present within the application-agnostic framework of a broad-scope measurement technique. The authors intend to present a detailed case-study in a separate publication, focused on analysis and application within a specific sub-field.

However, our virtual instrument is also based on a bulk of previous experimental data, both ours and others [1–4], and is therefore also deeply rooted in experimental data. As four of our authors are industrial authors, rooting our developments in verifiable experimental work was critical, and we have additionally added the following to include this information:

4 Modelling virtual parts

It has long been been established that, within the physical constrains of non-elastic and non-environmentally aged surfaces, there are a small number of parameters which define their shape. These parameters are commonly grouped into surface roughness and surface profile parameters (further broken down into runout and roundness). Since these physical features are those that cause runout and roundness deviations, it is these that have been aggregated to form the instrument, and that it aims to measure with the proposed 3D runout method. To keep applicability broad, no component-type specific (e.g. gear) features are considered, instead the components are kept as general cylinders with runout and roundness perturbations added to their shape. These perturbations cover the complete set of runout, roundness, and surface roughness features expected on physical parts from previous experimental work and known measuremnt protocols [3, 10, 23–27].

The virtual components themselves were defined on-grid; a 3D byte array was [illegible]

2.5 *Visual Presentation Figures and Tables: The format and style of figures and tables should be unified to maintain consistency throughout the paper. Additionally, optimising the layout of these visual elements could improve readability and the overall presentation quality.*

We thank the reviewer for spotting visual inconsistencies that we have missed and have now updated them to be self-consistent throughout the paper. We have also added the following figure, as per the suggestion of the reviewer, and would appreciate it if they could make sure we have been self-consistent with the additions as well:

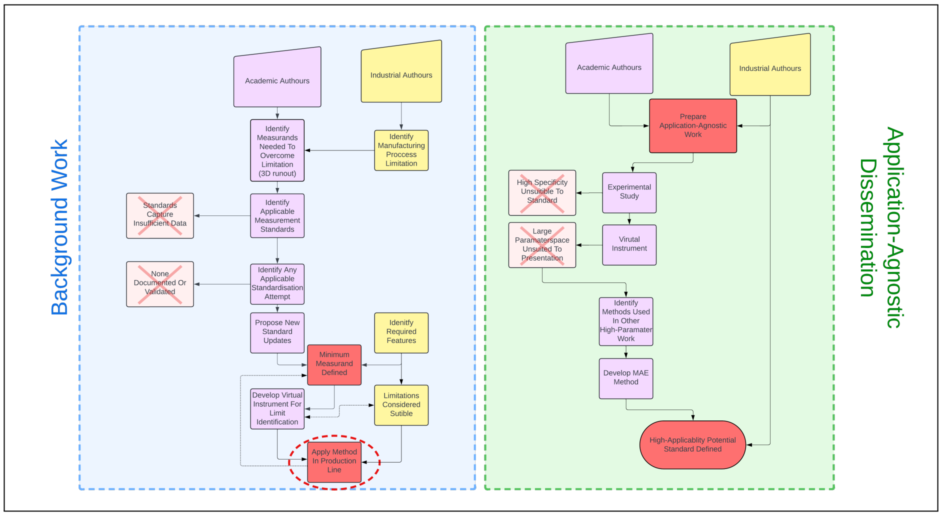

2.6 *Future Work Extension of the Method: The discussion on future work should elaborate on potential extensions of the proposed method. This might include adapting the method for different types of components, integrating it with other manufacturing processes, or enhancing it with machine learning techniques for improved accuracy and efficiency.*

As the reviewer rightly points out, there are a lot of potential future avenues for this, so we have chosen to mention two additional points: The first being the reviewers suggestion of AI adoption, as we found it a particularly interesting approach to future metrology and smart manufacturing, and it primarily is only possible because of the extra spatial information our method provides. The second point we included is the potential to measure runout on non-standard surfaces, such as a rate of change measurement on curved surfaces, as the extra spatial information would allow you to track feature changes as a part is milled (for example).

The model itself is also not limited to the linear cases modelled here, and can be extended to accounted for curved surfaces, much in the same way that the definition of polygonal meshes to quantise an object is used in CAD and FEM scenarios. This method could be extended to higher fidelity as hardware and computational methods advance, allowing the basic method to be retained permanently, even on more complex and non-linear geometries as computational analysis methods advance. Analysing a rate of change of runout in this case would be ideal when components need to be fitted together, but pose an interesting challenge for part selection. Integrating machine learning methods, trained via our MAE method, into the runout measurement process could provide the necessary computational intelligence required to improve the accuracy and speed

20

of our runout method to reach such levels of smart optimisation.

References

1. Yu, N. & Polycarpou, A. A. Contact of Rough Surfaces With Asymmetric Distribution of Asperity Heights. *Journal of Tribology* **124,** 367–376. ISSN: 0742-4787 (July 2001).
2. Molnar, V. Asymmetric Height Distribution of Surfaces Machined by Hard Turning and Grinding. *Symmetry* **14.** ISSN: 2073-8994 (2022).
3. Elerian, F. & Abou Eleaz, M. Methods of Roundness Measurement: An Experimental Comparative Study (Aug. 2021).

4. Yu, N. & Polycarpou, A. Combining and Contacting of Two Rough Surfaces with Asymmetric Distribution of Asperity Heights. *Journal of Tribology-transactions of The Asme - J TRIBOL-TRANS ASME* **126** (Apr. 2004).

3 Reviewer 3

The paper presents a novel method that accurately characterises the three-dimensional runout of rotation ally symmetric components using minimal surface information. Additionally, a new technique for assessing simulated errors in virtual instrument measurements is developed. Overall, the quality of the manuscript needs to be substantially improved, and the contributions are not strong enough.

We thank the reviewer for their time, and their appreciation of the novelty of our potential new runout analysis method, as well the techniques we developed to analyse new methodologies like it. The feedback has helped us identify some key limitations in our structure which hindered the perception of the scope of our work. Particularly, the reviewer helped us realise that we had not given enough context to our work, both in the general background and how our method was developed. In the sections below, the reviewer will find the itemised changes they have suggested, addressing these issues. We hope that these changes meet the reviewers expectations and they feel the novelty of our work will now be clear to the engineering-wide audience we intended to target with our work.

3.1 *The paper fails to adequately explain the practical background and value of the research, lacking a thorough explanation of why there is a need for rapidly characterising the three-dimensional runout of cylindrical components and its potential applications in engineering or manufacturing fields.*

The proposed method is applicable to different parts from gears t9o bearings, and pistons, so give specific details can be difficult. e. We believe they will also agree that their involved fields and needs are rapidly evolving in industry 4.0. It was our concern that high detail on one topic would give the impression it was only (or mainly) applicable there, and get overlooked in the other rapidly changing fields. The reviewer can understand how a lot of detail on a sub-field different to your own can cause people to overlook work in this way. For this reason, we feel that this work is best represented as application-agnostic, so that it might be broadly uptaken. Instead, we feel that high-specificity applications have value in a publication by themselves and that a far more detailed and application-specific background review is very relevant there. Both the industrial and academic authors of this paper are working together on a follow-on case study for a second publication, due to the success we have had in applying the work presented here. For the more broad background, we have included the following which explains the background of the current measurement standards

and their general limitations in more detail:

> The current 1D standards of roundness measurement have remained effectively unchanged since their analogue implementation 1985, before current digital analysis methods became widespread or affordable. Their limitations transferred over from by-hand methods and have been maintained for ease of use and tractability. However, technological advancements within the current automation/industry 4.0 framework mean that extending these standard beyond simple magnitude measurements, maintaining backwards compatibility and using the already increasing computation of (now smart) manufacturing, has now become both feasible and desirable. An addition of more physically representative information in the measurement, kept concise, would provide more knowledge of the measureand and allow for techniques like smart pairing of components using available machinery, instead of resource-heavy retooling/rejection. Outlined in this paper
>
> 2
>
> is, to the authors knowledge, the first proposed extension to these standards: a fully backwards-compatible extension which characterises runout in 3D, as a single-valued runout vector. This new method is tested and characterised to, determine the limits of the physical features it can measure, using another modern manufacturing tool: a virtual instrument.

We also realise that ”cylindrical” was potentially an inaccurate word to use, and implies the kind of specificity we intended to avoid. We have instead altered the discussion throughout to focus on ”rotationally symmetric” components to prevent this for now, but would welcome a more concise term if the reviewer has encountered a better one.

3.2 *Although a new method for defining and measuring three-dimensional runout is proposed, the paper lacks direct comparisons with existing methods, failing to demonstrate its innovativeness. No mention is made of similar studies, nor is the method's superiority over existing ones explicitly stated.*

The reviewer made a crucial point here, and one we did not adequately explain in the body of the manuscript. Part of the novelty of our work is that there are no equivalent standardised methods are able to gather spatial information about the runout of parts, which our industrial authors first realised as critical in the manufacturing of their parts. This lack of other methods is what caused them to approach the academic authors and develop this new method together. We have explained the workflow behind our developments by including a new workflow diagram (also included below). This diagram shows the development behind the work we present in this manuscript, and how we transferred it from commercially sensitive and highly specific work to something more broadly applicable. With the afforementioned additional explanation of the current measurement standards that we included thanks to the reviewer, we hopefeels we have included sufficient detail to give the readers context of the situation.

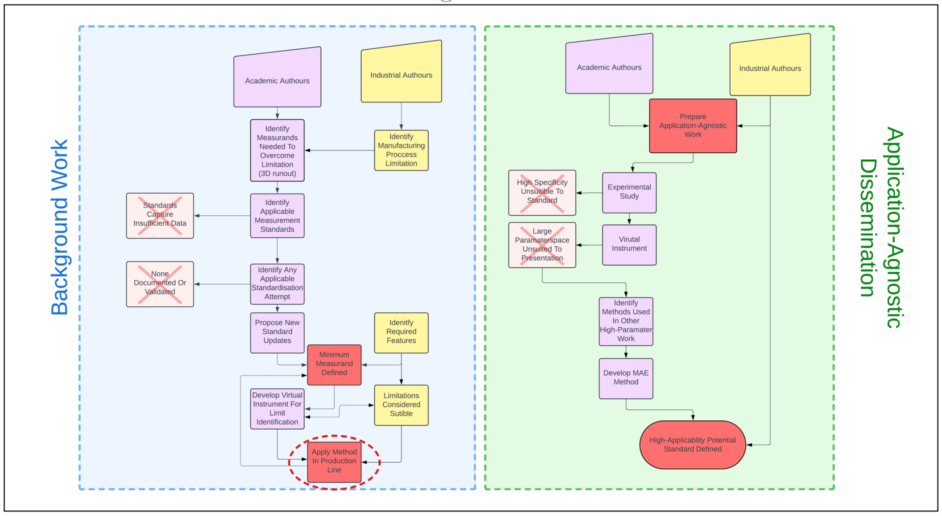

Figure A.1: The development pipleline that led to the work outlined in this manuscript. The initial ”background” workflow was a commercially-sensitive collaboration between the academic and industrial authors. This was streamlined and developed into the application-agnostic work presented here. One element, circled in red, was not replicated during the transfer. This was a high-specificity case study and deemed unsuitable to present within the application-agnostic framework of a broad-scope measurement technique. The authors intend to present a detailed case-study in a separate publication, focused on analysis and application within a specific sub-field.

3.3 *While virtual instruments are used for simulated experiments in the paper, no comparisons are made with existing methods or standard measurement techniques. There is a lack of sufficient experimental data to assess the performance and accuracy of the new method.*

We agree with the reviewer that a high-specificity experimental study is valuable, and four of our authors are industrial authors from a multi-national manufacturing company, so for us practical applicability was critical. The method we present was developed in tandem with a commercially-sensitive case study for this reason. This case study was not replicated in this manuscript because we are hopeful it can form the basis of a new standard. As the reviewer will know well, these are application-agnostic, and so is the initial ”method discovery” work. It is this stage of a standard development that we feel our work is at, and we wanted to target a broad audience in a similar way to these early standardisation-stages, and so did not replicate the case study and restructured our work accordingly. Instead, we intend to replicate our case study in a separate publication which is very heavily targeted on the applications of our method to a specific sub-field and the application-specific challenges that only one application faces. We have added these clarifications that reviewer helped us identify to the workflow diagram as well, so that the reader can understand the choices without breaking the flow of the main text.

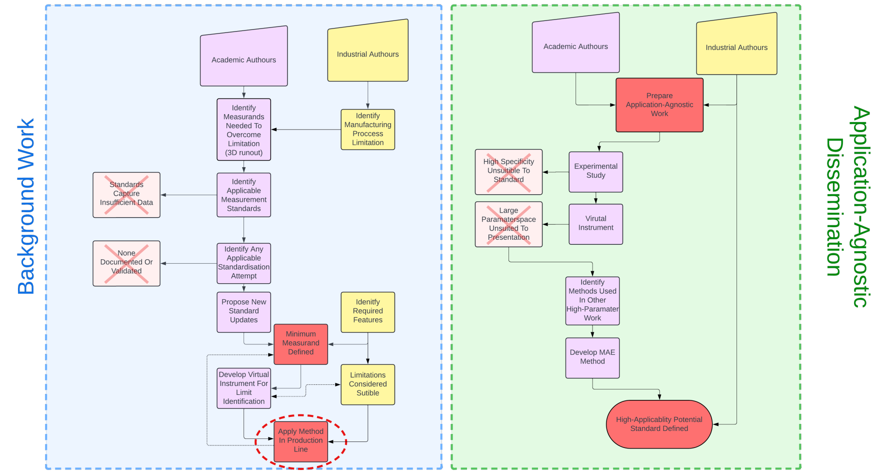

Figure A.1: The development pipleline that led to the work outlined in this manuscript. The initial "background" workflow was a commercially-sensitive collaboration between the academic and industrial authors. This was streamlined and developed into the application-agnostic work presented here. One element, circled in red, was not replicated during the transfer. This was a high-specificity case study and deemed unsuitable to present within the application-agnostic framework of a broad-scope measurement technique. The authors intend to present a detailed case-study in a separate publication, focused on analysis and application within a specific sub-field.

3.4 *The interpretation of experimental results is abstract, lacking specific data support and quantitative analysis. In discussing the impact of key parameters on the results, insufficient experimental data and analysis are provided, leading to a lack of credibility and comprehensibility in the conclusions*

The way in which we presented our results was a difficult development point, as we needed to present a five-dimensional parameter space in a tidy way, and the reviewer will understand that doing this on a 2D page is not possible. Instead, we took inspiration from the large body of other engineering and AI work that uses large parameter spaces, where similar problems exist. We feel that the methods in these papers [1–4], where they measure how well an analysis method works by comparing an output to what an output should be and recording the conformance value, to be applicable to our work. In our case, a virtual object is our output and the initial virtual object is what we are comparing it against.

These are conformant if analysis method has captured all the relevant defining parameters, with the conformance value being measured by the MAE. However, we have additional errors due to features like noise, where equivalent surfaces are not necessarily identical, and this understandably means we have an acceptance threshold. We determined this threshold to be 0.5%, with any value greater than this meaning a parameter has changed.

Of course, the work we have presented here is open access and the data available. If the reviewer feels it would be more valuable to include a complete set of graphs, a few hundred in total, then we are happy to add this to the repository as well. For now, we have instead altered the text to include the following description of the parameter space and how the analysis method navigates this presentation-based problem:

ment accuracy. In this case, features may be a combination of part and mandrel parameters, such as a tilted part on a tilted mandrel .

As there is a five-dimensional data-space, the conformance of each individual parameter cannot be directly plotted in any concise manner [17–20]. For this reason the mean absolute error (MAE) of the residuals between input (I) and output (O) datasets are calculated following works by [17–20]:

$$MAE = \frac{\sum_i^n |I_i - O_i|}{n\overline{I}}, \tag{4}$$

where i is each element within the set. In this case, the datasets are profiles scans of a generated surface with the parameters: tilt, part offset, lobe amplitude, lobe count, and mean asperity height. Two profiles are therefore not identical unless all parameters which define them are identical. To generate the two sets of profiles, 3D part-mandrel assemblies are generated and analysed with the virtual instrument, one with the input parameters (I), and one with the output parameters (O). The input parameters are user defined, and the output parameters were obtained by scanning the initial part and then recovering the runout/roundess parameters with the proposed 3D runout model, then using these to generate a new part. This new part is then measured, to generate new

profile scans for comparison.

References

1. Qi, J., Du, J., Siniscalchi, S. M., Ma, X. & Lee, C.-H. On Mean Absolute Error for Deep Neural Network Based Vector-to-Vector Regression. *IEEE Signal Processing Letters* **27,** 1485–1489. ISSN: 1558-2361 (2020).
2. Error Metrics and Performance Fitness Indicators for Artificial Intelligence and Machine Learning in Engineering and Sciences. eng. *Architecture, Structures and Construction* **3,** 499–517. ISSN: 2730-9886 (2023).
3. Wang, Z., Bovik, A., Sheikh, H. & Simoncelli, E. Image quality assessment: from error visibility to structural similarity. *IEEE Transactions on Image Processing* **13,** 600–612 (2004).
4. Yuan, S., He, Z., Zhao, J., Yang, Z. & Yuan, Z. Long-term electrocardiogram signal quality assessment pipeline based on a frequency-adaptive mean absolute deviation curve. eng. *Applied intelligence (Dordrecht, Netherlands)* **53,** 20418–20440. ISSN: 0924-669X (2023).

Reviewer Replies to "Three-Dimensional Runout Characterisation For Rotationally Symmetric Components V2"

August 22, 2024

1 Reviewer 1

We are pleased that the changes we made mostly satisfied the reviewer and have addressed their remaining point below, hopefully also to their satisfaction, and are glad that they then feel our work is ready for publication.

1.1 An experiment setup structure principle and photo should be provided at least.

We follow the reviewers line of thinking, and we have added this to our first figure to help give some more context. This image gives the set-up for the visualised experiment, which could then be translated to physical hardware. A copy is shown below:

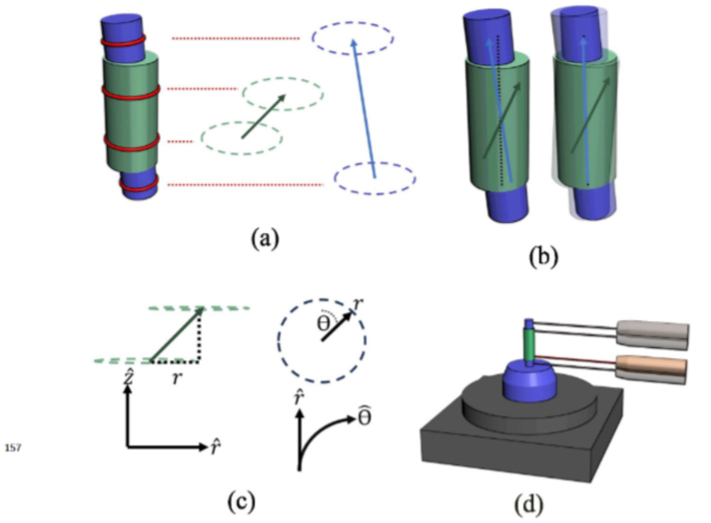

Figure 1: The proposed method of defining three-dimensional runout. In (a), the part in question (green) is mounted on a mandrel (blue), about which it is rotated. The four measurement positions are highlighted in red. The runout vectors of each component are then visualised. In (b), the runout of the mandrel is subtracted from the part, leaving the runout of the part defined only in the coordinate axes. silhouettes of the assembly have been added to illustrate the transform. (c) shows how the runout vector is defined in the coordinate axes ($\hat{r}$ and $\hat{\theta}$), with magnitude r and direction θ. $\hat{z}$ is the final direction which defines the cylindrical polar coordinate system, and is normal to the plane of rotation. Finally, in (d), an example of the potential hardware set-up for this is shown. The part and mandrel are now mounted on a rotation stage (grey), with a sensor (orange) directed at one of the four measurement zones, with the remaining 3 zones silhouetted.

2 Reviewer 2

We are pleased that, aside form some minor points, the reviewer feels our work is ready for publication, and thank them for their input, which helped get it to that point. The reviewer will find our replies to their remaining concerns below

2.1 1) The new ”method summary” section is a valuable addition. However, it could be further refined to succinctly highlight the method’s key steps and advantages. Besides, why the development pipeline was provided in the appendix, instead of in Section 2? What’s more, where is A.2 if the Appendix includes A.1?

We have updated our method summary description to include the extra detail, copied in below. For the new diagram, we chose to add this pipeline in the appendices instead of the main body, since we felt is was quite a large figure and some readers may wish to dive straight into the method details when they realise they have an application for it. For those that are more interested in the development process behind it and its wider application, we imagined they would not mind reading an appendix. Of course, we can always move this, if that is preferred. For the appendix numbering, we are using the default ”Nature” referencing style in our LaTeX document and haven’t altered it, although we have now added another appendix figure, based on the input of another reviewer.

> The first methodology section in this work details the new proposed 3D runout method, listing the measurement process and how the 3D measure is produced from four ISO-complaint measurements by defining a set of runout vectors. The section also proceeds to explain the new relevant measurement quantities that other methods cannot measure: part orientation, part tilt, and runout direction. Following the 3D runout definition, a virtual instrument is presented.

2.2 2)Providing examples of potential applications and their theoretical underpinnings would help illustrate the method’s broader impact.

We have provided some additional cited examples of applications, including a review article which goes quite deeply into the theory behind data-driven smart manufacturing in a wide range of scenarios, and the current data limitations which techniques like ours may address:

retooling/rejection. Recent reviews of the current smart-manufacturing landscape highlight how current methodologies may not be suited to automated processes such as this. They instead require more data driven approaches to allow for proper machine control, instead of the intuitive leaps humans may make, but with potentially significant decreases in financial/time cost in implementing them [15]. Of course, maintaining tractability with these new techniques then becomes an important challenge - the new methods must be compatible with the old. Outlined in this paper is, to the authors knowledge, the first proposed

2.3 3)Detailed explanation of how the proposed method compares to existing methods in terms of accuracy, reliability, and applicability would be appreciated.

This is an interesting point and one of the key reasons we designed our method to be a backwards-compatible extension of the current methods. In the cases where current methods can be used, our method is identical and therefore any measurement taken with our method is consistently traceable. We know the reviewer will understand the importance of consistent tractability and believe they will agree that extending a method to maintain this is more trustworthy than completely new method. We have added the following clarifications to the text to highlight how this compatibility is maintained:

a parts fixturing. This runout model extends the standard definition of runout [7] by giving it magnitude and direction – a runout vector. By extending the ISO-compliant methods in this way, combining multiple already standardised measurements to produce a vector with additional data, all currently recoverable measurable features may be measured with the same accuracy and reliability. Traceability is, crucially for upgrading facilities, therefore fully maintained by this runout vector. This vector is defined in cylindrical polar coordinates, giving a

3 Reviewer 3

We are pleased that, with the decision as to whether we include graphs in the repository (that we left to the reviewers expertise), there was only one remaining point that the reviewer felt was hindering our work. We thank them for their improvements so far, and hope this final point is sufficiently addressed in the changes below.

3.1 Evaluation: The authors provided a detailed explanation of their decision to keep the application general and added broader background information. These modifications significantly improved the explanation of the study's background. I agree with the authors that high-detail applications can be published in subsequent specific studies. Therefore, I am satisfied with the changes made to this section.

We are glad these changes were satisfactory and thank the reviewer for suggesting the improvements we made.

3.2 Evaluation: The authors' response and the new workflow diagram effectively explain the uniqueness of their method and its development background. These additions successfully demonstrate the method's innovativeness and provide the necessary context. I find this response and the modifications satisfactory.

As above, we are glad these are satisfactory and thank the reviewer.

3.3 Evaluation: The authors' explanation is reasonable and clearly outlines why they did not include specific case studies. Their plan to detail the practical applications in future studies is appropriate. However, the current manuscript still lacks experimental data and comparative analysis, which are crucial for assessing the new method's performance. I suggest adding some key experimental data and comparisons in the current manuscript to strengthen its credibility and completeness.

Including experimental data while keeping the work agnostic, as agreed, is challenging but we believe the graph we have included below should satisfy this. We have added a figure and description but included it in the appendix, since it is

preliminary and will form part of the validation steps in our next paper. In this figure some simple validation artefacts were manufactured and then measured with one of our measurement instruments to produce profile scans, in-line with our method. These artefacts were manufactured cylinders, where a known runout (tilt) was manufactured into the design, at a far larger value that the tolerance of either the equipment or measurement hardware. Following our measurement process and passing the data through our 3D runout analysis script should then recover the manufactured tilt, if our method is valid. This was found to be the case the case for the test artefacts, as the reviewer will conclude form the graph. As this is one of the key new measurable features of our method, and these simplistic parts do not require the complex manufacturing steps that start to fall into case-study territory, we hope the reviewer feels this is an appropriate inclusion to fit their request for some experimental indication.

A.2 Experimental Sample Data

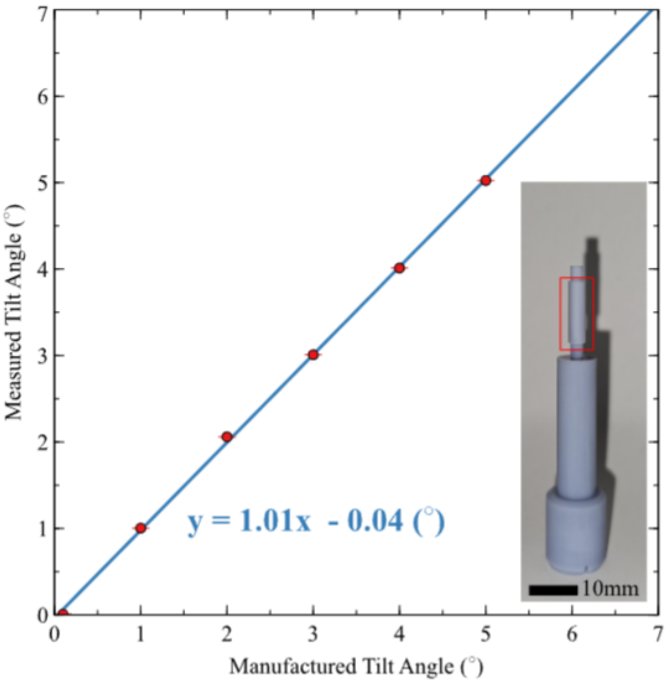

Figure A.2: Example experimental data of a validation artefact measured with the method outlined in this work. Four measurements are taken along the artefact, and post-processed with the 3D runout method. Compared are the manufactured angles of 5 artefacts, and the angle recovered using the 3D runout method, showing Inset is an image of the validation artefact, with scale bar. The region within the red bounding box is a cylinder, manufactured with a known tilt angle, the "part". The remainder in the "mandrel", with the four measurement zones selected using the method outlines in this work. Remaining features are registration marks.

3.4 Evaluation: The authors' response and modifications improved the explanation of the parameter space and provided a clearer analysis method. However, to enhance the paper's credibility, I suggest including more specific experimental data and graphs in the main text, even if linked to an appendix or online repository, to significantly improve the paper's credibility and comprehensibility.

In our previous reply to this reviewer, we mentioned that we were happy to prepare the few hundred or-so graphs of individual comparisons and include them in the repository, if they felt this would be more valuable than just the raw data. Based on their response here, we see that including all of these is the reviewer's preference, and therefore will include them. The following addition has been made to the main text to also make this inclusion clear to the readers:

> As there is a five-dimensional data-space, the conformance of each individual parameter cannot be directly plotted in any concise manner, although these may also be found in the data repository for individualised inquiries [17–20]. For this reason the mean absolute error (MAE) of the residuals between input (I) and output (O) datasets are calculated following works by [17–20]:
>
> $$MAE = \frac{\sum_i^n |I_i - O_i|}{n\bar{I}}, \quad (4)$$

Reviewer Replies to "Three-Dimensional Runout Characterisation For Rotationally Symmetric Components V3"

October 8, 2024

No reviewer changes were recommended for this version - thank you all for your time.
